# A Physics-Informed Benchmarking Framework for Machine Learning and Tree-Based Ensembles in IIoT-Enabled Predictive Maintenance

**DOI:** 10.3390/s26165026

**Published:** 2026-08-07

**Authors:** Yi-Kai Su, Chun-Jan Tseng

**Affiliations:** 1 Department of Financial Management, National Defense University, Taipei 112305, Taiwan; ndmcc10003@gmail.com; 2 Department of Data Science, Soochow University, Taipei 111002, Taiwan

**Keywords:** Predictive Maintenance (PdM), Industrial Internet of Things (IIoT), physics-informed machine learning, engineering-guided feature engineering, class imbalance, tree-based ensembles

## Abstract

Reliable Predictive Maintenance (PdM) in Industrial Internet of Things (IIoT) environments is challenged by severe class imbalance, heterogeneous sensor variables, inconsistent experimental protocols, and deployment constraints. This study proposes a Physics-Informed Benchmarking Framework that integrates engineering-guided feature construction, Mutual Information (MI)-based feature relevance analysis, standardized model development, and deployment-oriented evaluation within a unified and reproducible workflow. Using the AI4I 2020 Predictive Maintenance Dataset, Logistic Regression, Isolation Forest, Random Forest, and Extreme Gradient Boosting (XGBoost) were evaluated using identical feature representations, train–test partitions, preprocessing procedures, and imbalance-handling strategies. The engineered feature space incorporates thermal, mechanical, interaction, and degradation-related information derived from the original sensor measurements. The results show that tree-based ensembles provide the strongest overall performance under severe class imbalance. Random Forest achieved an accuracy of 0.986, an F1-score of 0.722, and a ROC-AUC of 0.983, providing the best balance between failure detection and false-alarm control. XGBoost achieved an accuracy of 0.978, a recall of 0.853, and the lowest inference latency of 0.35 ms, indicating its suitability for latency-sensitive IIoT deployment. These findings demonstrate that combining engineering-guided feature representation with a standardized evaluation protocol enables fair comparison of representative learning paradigms while preserving engineering interpretability and deployment relevance.

## 1. Introduction

### 1.1. Manufacturing Paradigm Shift Toward Predictive Maintenance

The rapid advancement of the Industrial Internet of Things (IIoT), cyber–physical systems (CPS), edge computing, and artificial intelligence (AI) has transformed modern manufacturing from rule-based automation to intelligent, data-driven production [1]. In Industry 4.0 environments, industrial assets continuously generate heterogeneous sensor data that support real-time monitoring, diagnosis, and decision-making for manufacturing systems [2,3,4].

Against this background, Predictive Maintenance (PdM) has become a key strategy for improving equipment reliability and operational efficiency. Unlike reactive maintenance, which intervenes only after failures occur, or preventive maintenance, which relies on fixed service intervals, PdM continuously monitors equipment health using sensor data and predicts potential failures before severe degradation occurs [5,6]. This condition-based maintenance strategy reduces unplanned downtime, improves asset availability, lowers maintenance costs, and supports remaining useful life (RUL) estimation [7].

Machine learning (ML) has further accelerated the development of PdM by learning nonlinear relationships from multisource industrial sensor data. Previous studies have also demonstrated that incorporating domain knowledge into machine learning models can improve prediction performance in semiconductor manufacturing, such as the fusion network architecture proposed by Liu et al. [8]. Typical PdM variables include temperature, rotational speed, torque, vibration, acoustic emission, electrical current, and tool wear. Collectively, these measurements provide valuable information for anomaly detection, fault classification, and health assessment [9,10].

Despite these advances, practical PdM deployment remains challenging because industrial applications require not only high predictive accuracy but also computational efficiency, interpretability, and real-time responsiveness [8]. Models deployed in IIoT or edge computing environments must operate with limited computational resources while providing physically meaningful evidence to support maintenance decision-making [11,12]. Motivated by these industrial requirements, this study proposes a Physics-Informed Benchmarking Framework for highly imbalanced PdM using the publicly available AI4I 2020 Predictive Maintenance Dataset https://www.kaggle.com/datasets/stephanmatzka/predictive-maintenance-dataset-ai4i-2020 (accessed on 26 June 2026).

### 1.2. Challenges in Highly Imbalanced IIoT-Based PdM

Although ML-based PdM has been rapidly adopted, its practical deployment in industrial settings remains difficult because actual equipment failures are inherently rare. Industrial machinery is deliberately kept in healthy operating conditions for most of its lifetime, which produces highly imbalanced datasets where failure instances represent only a small portion of all observations. In such scenarios, standard learning algorithms are typically skewed toward the majority class, resulting in poor failure detection performance even when the overall classification accuracy appears high [9,13].

The issue of class imbalance is especially acute in PdM because the cost of undetected failures is far greater than the cost of false alarms. A classifier that labels most samples as normal may achieve seemingly high accuracy yet still miss critical impending faults. Therefore, deployment-focused PdM should prioritize evaluation metrics such as Precision, Recall, F1-score, and Receiver Operating Characteristic–Area Under Curve (ROC-AUC), instead of relying exclusively on overall classification accuracy [14,15].

A further challenge stems from the deployment context of contemporary IIoT infrastructures. Many PdM solutions run on edge or embedded industrial devices that are constrained in terms of computation, memory, and latency. While deep learning approaches have achieved strong predictive results, their heavy computational demands and limited transparency frequently hinder their use in real-world, resource-limited manufacturing settings [4,12,16].

Collectively, these issues show that effective PdM in practice requires more than highly accurate predictive models. Successful deployment also relies on physically interpretable feature representations, standardized benchmarking protocols, and computationally efficient learning methods that perform robustly under severe class imbalance in industrial data. Meeting these needs constitutes the primary motivation for the research conducted in this work.

### 1.3. Limitations of Existing PdM Approaches

Recent progress in ML and deep learning has greatly enhanced PdM effectiveness across diverse industrial domains. Typical methods include supervised learning for fault diagnosis, unsupervised techniques for anomaly detection, ensemble-based models, and deep neural networks tailored to capture complex degradation behaviors. When ample labeled data and sufficient computational power are available, these techniques have shown strong predictive performance [17,18,19].

Despite these achievements, several limitations remain. First, many existing studies emphasize model accuracy while paying relatively limited attention to the physical relationships among sensor variables. Raw sensor measurements are frequently adopted as direct model inputs without incorporating engineering knowledge regarding thermal behavior, mechanical loading, or degradation mechanisms. As a result, the learned feature representations often lack physical interpretability.

Second, comparisons among PdM models are frequently conducted under different preprocessing procedures, feature representations, sampling strategies, and evaluation criteria. Such inconsistencies make it difficult to objectively compare the effectiveness of different learning algorithms and reduce the reproducibility of published benchmark results.

Third, a growing body of recent work has concentrated on boosting predictive performance by employing increasingly advanced deep learning architectures. While such models frequently deliver very strong classification results, they typically demand considerable computational power, making them less appropriate for use in resource-limited IIoT and edge-computing settings [20]. Moreover, their black-box nature can undermine user trust in maintenance-related decision-making [21,22,23].

These observations reveal a broader research gap in deployment-oriented PdM. Although previous studies have reported improvements through advanced learning algorithms, feature engineering, or imbalance-handling techniques, these components are typically investigated in isolation and evaluated under different experimental settings. Consequently, it remains difficult to determine whether the reported performance gains arise from the learning algorithms themselves or from differences in feature representation, preprocessing procedures, and evaluation protocols. This lack of standardized and reproducible benchmarking limits objective comparison among existing PdM approaches and hinders their practical adoption in IIoT environments. Therefore, there is a clear need for a unified benchmarking methodology that integrates engineering-guided feature representation, standardized experimental protocols, and deployment-oriented evaluation within a single reproducible framework.

### 1.4. Proposed Physics-Informed Benchmarking Framework

To address the research gap identified in the preceding section, this study proposes a Physics-Informed Benchmarking Frameworkfor deployment-oriented predictive maintenance. Unlike conventional PdM studies that primarily optimize individual components of the learning pipeline, such as feature engineering, model architecture, or data imbalance handling, the proposed framework integrates these complementary aspects into a unified and reproducible experimental methodology. Rather than introducing another prediction algorithm, the framework establishes a standardized benchmarking workflow that combines engineering-guided feature construction, feature relevance validation, consistent model evaluation, and deployment-oriented performance assessment. This integrated design enables objective comparison among representative machine learning approaches while simultaneously considering predictive accuracy, engineering interpretability, computational efficiency, and practical deployment requirements.

**Figure 1:**
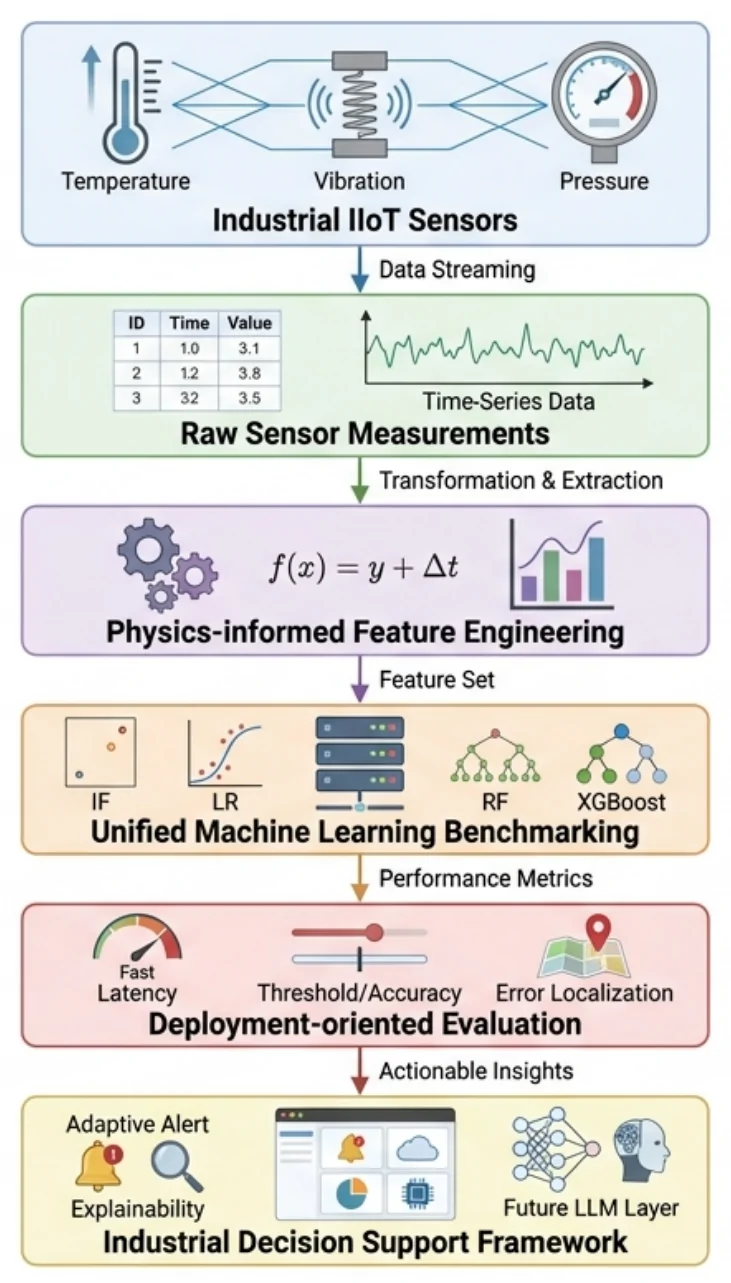
Overall architecture of the proposed Physics-Informed Benchmarking Framework. The framework integrates physics-informed feature engineering, feature relevance analysis, benchmark model development, and deployment-oriented evaluation into a unified workflow for industrial PdM.

Figure 1 illustrates the overall workflow of the proposed framework. Starting from the AI4I 2020 Predictive Maintenance Dataset, the framework first conducts exploratory data analysis to identify physically meaningful relationships among multi-sensor signals. Engineering knowledge is then incorporated to construct physics-informed features that characterize thermal behavior, mechanical loading, degradation progression, and operational interactions. The resulting feature space is quantitatively validated using Mutual Information before representative lightweight ML algorithms are benchmarked under identical experimental conditions.

A key characteristic of the proposed framework is that engineering-guided feature representation precedes model development. Unlike conventional benchmarking studies that directly compare learning algorithms using raw sensor measurements, all candidate models in this study share the same preprocessing procedures, feature representations, imbalance-handling strategy, and evaluation metrics. This standardized experimental protocol minimizes methodological bias and enables a fair, transparent, and reproducible comparison of representative learning algorithms. All benchmark models share the same preprocessing steps, feature set, imbalance-handling methods, and evaluation metrics, enabling fair comparison and improving reproducibility.

Beyond conventional classification accuracy, the proposed framework adopts a deployment-oriented evaluation strategy that jointly considers predictive performance, minority-class detection capability, computational efficiency, engineering interpretability, and industrial applicability. Consequently, model selection is guided not only by predictive performance but also by practical deployment considerations relevant to resource-constrained IIoT environments. This evaluation philosophy provides a more comprehensive basis for selecting lightweight PdM solutions for real industrial applications [24].

As illustrated in Figure 1, the proposed methodology differs from conventional PdM workflows in two fundamental aspects. First, engineering knowledge is incorporated before model development through the construction of physics-informed features rather than relying solely on raw sensor measurements. Second, all benchmark models are evaluated under an identical experimental protocol, ensuring that observed performance differences primarily reflect algorithmic characteristics instead of inconsistencies in preprocessing or evaluation procedures. Consequently, the proposed framework establishes a reproducible benchmarking methodology that bridges sensor-level data analytics with practical IIoT deployment while preserving engineering interpretability throughout the PdM process.

### 1.5. Major Contributions

The primary scientific contributions of this study are summarized as follows:

1. A unified Physics-Informed Benchmarking Framework is proposed for deployment-oriented PdM. Rather than introducing a new prediction algorithm, the framework establishes a standardized and reproducible experimental methodology that integrates engineering-guided feature construction, feature relevance validation, benchmark model development, and deployment-oriented evaluation. This unified workflow enables objective comparison of representative ML algorithms while improving benchmarking transparency and experimental reproducibility.
2. A set of physics-informed engineering features is developed by incorporating domain knowledge of thermal behavior, mechanical loading, degradation progression, and operational interactions into heterogeneous IIoT sensor measurements. These engineered features enhance feature representation by embedding engineering knowledge into the learning process, thereby improving both predictive capability and engineering interpretability without increasing model complexity.
3. A standardized benchmarking protocol is established in which representative lightweight ML algorithms, including Logistic Regression, Isolation Forest, Random Forest, and Extreme Gradient Boosting (XGBoost), are evaluated under identical preprocessing procedures, feature representations, imbalance-handling strategies, and evaluation metrics. This protocol minimizes methodological bias and enables fair, transparent, and reproducible comparison among different learning paradigms.
4. A comprehensive deployment-oriented evaluation methodology is developed by jointly assessing predictive performance, minority-class detection capability, computational efficiency, engineering interpretability, and industrial applicability. The proposed evaluation strategy provides practical guidance for selecting lightweight PdM solutions according to real-world IIoT deployment requirements rather than relying solely on predictive accuracy.

### 1.6. Organization of the Paper

The rest of this paper is structured as follows. Section 2 reviews recent progress in PdM, physics-informed machine learning, feature engineering, and benchmarking methodologies. Section 3 introduces the proposed Physics-Informed Benchmarking Framework, detailing the experimental workflow, dataset, feature engineering strategy, benchmark model development, and deployment-oriented evaluation protocol. Section 4 presents the experimental findings, feature relevance analysis, benchmark comparisons, deployment-focused assessment, and engineering insights. Finally, Section 5 synthesizes the main conclusions, discusses the limitations of this work, and highlights directions for future research.

## 2. Literature Review

### 2.1. Predictive Maintenance in IIoT-Enabled Manufacturing

**Figure 2:**
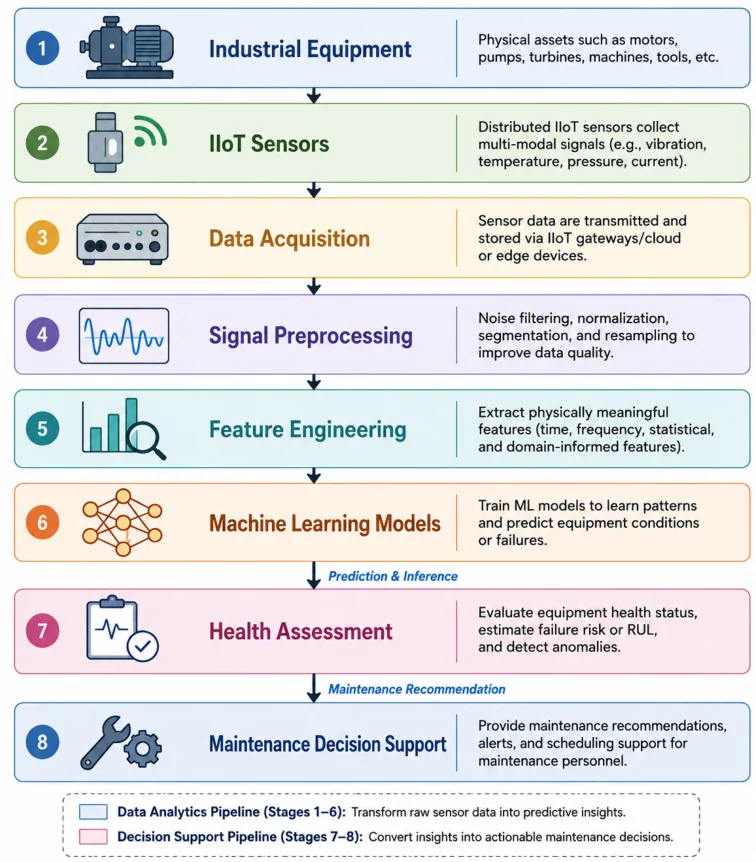
General workflow of an IIoT-enabled PdM system. Industrial equipment is continuously monitored through distributed IIoT sensors, followed by data acquisition, signal preprocessing, feature engineering, ML-based health assessment, and maintenance decision-making. This workflow represents the common PdM pipeline reported in the literature and serves as the conceptual foundation of the proposed framework.

Recent progress in IIoT technologies has greatly broadened the use of PdM in contemporary manufacturing. Through the continuous acquisition of diverse sensor data from industrial machinery, IIoT infrastructures support data-driven condition monitoring, fault detection, health evaluation, and maintenance planning. As a result, PdM has shifted from standalone fault prediction to a comprehensive engineering decision-making framework that integrates sensing, feature engineering, ML, and maintenance scheduling [25,26]. Figure 2 depicts the typical workflow employed in IIoT-based PdM systems.

As shown in Figure 2, PdM is best understood as a comprehensive, end-to-end analytical workflow rather than a standalone classification problem. The effectiveness of real-world PdM solutions is jointly shaped by sensor data quality, feature engineering, learning algorithms, and maintenance decision-support mechanisms. As a result, recent research has placed growing emphasis on embedding engineering knowledge across the entire analytical pipeline, instead of concentrating exclusively on model development.

Despite these advances, existing PdM studies differ substantially in feature representation, benchmark design, and deployment considerations. The following sections therefore review recent developments in physics-informed feature engineering, representative ML paradigms, and deployment-oriented benchmarking methodologies [27,28,29].

### 2.2. Physics-Informed Feature Engineering for PdM

Feature engineering is a cornerstone of PdM, as the predictive power of ML models is heavily influenced by how relevant and physically meaningful the input variables are [30]. While recent deep learning methods can automatically learn features from raw sensor streams, many real-world PdM systems in industry still depend mainly on structured sensor readings rather than large-scale time-series data. As a result, manually derived features remain prevalent due to their computational efficiency, engineering interpretability, and suitability for lightweight ML models [9,19].

In contrast to purely statistical feature extraction, the proposed feature engineering strategy embeds engineering knowledge directly into the feature construction process by explicitly accounting for the physical interdependencies among sensor signals. Instead of viewing each sensor reading as an isolated numerical value, physically interpretable transformations are devised to more accurately represent machine operating states and underlying degradation processes. Typical examples include temperature differentials, accumulated tool wear, rolling-window statistics, load fluctuations, vibration energy, frequency-domain descriptors, and interaction terms constructed from multiple sensors. These engineered variables frequently yield stronger predictive performance and more intuitive physical insight than raw measurements alone.

From an industrial standpoint, engineering-guided feature construction provides several tangible benefits. First, incorporating engineering knowledge into the feature space simplifies subsequent learning tasks. Second, physically interpretable variables enhance model transparency, allowing maintenance engineers to relate model outputs to established engineering concepts. Third, such engineered features tend to be more resilient to measurement noise and support the transfer of knowledge across different ML models, thereby enabling standardized benchmarking within a common feature representation.

Recent studies have demonstrated that carefully designed feature engineering can substantially improve PdM performance and, in some industrial applications, may provide benefits comparable to those obtained through incremental improvements in learning algorithms [31,32]. Rather than relying exclusively on increasingly complex models, many successful industrial applications have achieved substantial performance improvements by combining domain knowledge with statistical feature transformation and feature selection techniques [33,34]. These findings indicate that feature engineering should be regarded as an integral component of PdM system design rather than merely as a preliminary data-processing step.

Although feature engineering and feature selection are closely related, they address different objectives. Feature engineering enriches the feature space by constructing physically meaningful variables, whereas feature selection identifies the most informative subset by removing redundant or irrelevant features. Existing studies often investigate these two processes independently, while comparatively limited attention has been devoted to integrating engineering-guided feature construction with systematic feature relevance evaluation within a unified PdM framework.

Therefore, there remains a need for a reproducible methodology that combines physics-informed features with standardized feature relevance analysis and benchmark evaluation. Such a framework can simultaneously improve predictive capability, engineering interpretability, and experimental reproducibility for deployment-oriented PdM.

### 2.3. Machine Learning Algorithms for Predictive Maintenance

ML has become one of the dominant technologies for PdM because it enables complex relationships between multisource sensor measurements and equipment degradation to be learned directly from historical operating data. Compared with conventional rule-based diagnostic systems, ML algorithms provide greater flexibility in modeling nonlinear degradation behavior and can effectively utilize large volumes of industrial monitoring data. Depending on the availability of labeled failure information and application requirements, PdM methods can generally be categorized into supervised learning, unsupervised anomaly detection, and deep learning approaches [35,36,37].

Commonly adopted supervised algorithms include Logistic Regression (LR), Support Vector Machines (SVMs), Decision Trees, Random Forest (RF), and XGBoost. These models have demonstrated competitive predictive capability while maintaining relatively low computational complexity, making them suitable for industrial applications where model efficiency and interpretability are important considerations [17,18].

Among these approaches, LR, RF, and XGBoost are selected as representative supervised benchmark models in this study because they exhibit complementary learning characteristics. LR provides an interpretable linear baseline, whereas RF and XGBoost represent two widely adopted tree-based ensemble strategies for modeling nonlinear relationships. RF reduces variance through bootstrap aggregation, while XGBoost sequentially optimizes weak learners using gradient boosting. Tree-based ensembles are generally robust to noisy sensor measurements, accommodate heterogeneous feature types, and provide feature-importance information that can support engineering interpretation. These characteristics have contributed to their widespread adoption in industrial PdM [38,39].

When labeled failure data are unavailable or severely limited, unsupervised learning provides an alternative solution by identifying abnormal operating conditions without explicit failure labels. Typical methods include Isolation Forest (IF), Local Outlier Factor, and One-Class Support Vector Machines. Rather than directly learning failure characteristics, these algorithms identify observations that deviate significantly from normal operating behavior. Although unsupervised approaches are attractive for practical industrial environments where failure events are rare, their predictive performance generally depends strongly on the definition of normal operating conditions and may be inferior to supervised learning when sufficient labeled failure data are available [40].

Recent studies have increasingly explored deep learning architectures for PdM, including Convolutional Neural Networks (CNNs), Long Short-Term Memory networks (LSTM), Transformers, and hybrid neural models. These approaches automatically learn hierarchical feature representations from high-dimensional sensor signals and have demonstrated excellent predictive performance for large-scale industrial datasets. Nevertheless, their substantial computational requirements, dependence on extensive training data, and limited interpretability may restrict their practical deployment in IIoT and edge-computing environments where computational resources are constrained [20,21,22,23]. Accordingly, deep learning models were not included as benchmark algorithms in this study because the primary objective is to establish a standardized and reproducible benchmarking framework using representative machine learning paradigms that are widely adopted in industrial PdM. The proposed framework is, however, algorithm-independent and can be readily extended to incorporate deep learning models in future investigations. In addition, the AI4I 2020 Predictive Maintenance Dataset contains approximately 10,000 tabular samples rather than large-scale sequential sensor streams. Under these conditions, representative conventional machine learning algorithms provide an appropriate basis for standardized benchmarking, whereas the advantages of deep learning architectures are generally more evident in large-scale time-series datasets with substantially richer temporal information.

Table 1 summarizes representative learning paradigms that are widely adopted in industrial PdM. Rather than exhaustively reviewing individual algorithms, the comparison highlights their learning characteristics, practical strengths, primary limitations, and deployment suitability. The algorithms selected in this study span linear, tree-based ensemble, and unsupervised learning paradigms, thereby providing a diverse but computationally practical basis for the standardized benchmark comparison presented in Section 3.

As outlined in Table 1, each learning paradigm offers distinct strengths and limitations depending on the availability of labeled failure data, computational resources, and target deployment constraints. In particular, tree-based ensemble methods are frequently adopted as strong benchmark models for PdM because they can capture nonlinear degradation patterns while maintaining comparatively favorable robustness, computational practicality, and interpretability. Regardless of the selected learning paradigm, model performance remains strongly influenced by feature representation and class imbalance. Consequently, imbalance-aware learning has become a central consideration in contemporary PdM research. As they can model nonlinear degradation patterns while preserving comparatively strong robustness and practical interpretability for engineers. However, irrespective of the chosen learning paradigm, model performance is still heavily affected by the quality of feature representations and the presence of class imbalance. As a result, imbalance-aware learning approaches have become a core element of contemporary PdM research.

Despite continuous advances in learning algorithms, class imbalance remains one of the most challenging problems in PdM because equipment failures occur much less frequently than normal operating conditions. Conventional ML algorithms trained on highly imbalanced datasets often become biased toward the majority class, resulting in poor minority-class detection despite achieving high overall classification accuracy [13,14]. Consequently, numerous imbalance-aware learning strategies have been proposed, including synthetic over-sampling, class weighting, cost-sensitive learning, and ensemble-based approaches. Among these methods, the Synthetic Minority Over-sampling Technique (SMOTE) remains one of the most widely adopted preprocessing techniques because it effectively improves minority-class representation while maintaining relatively low implementation complexity [41].

**Figure 3:**
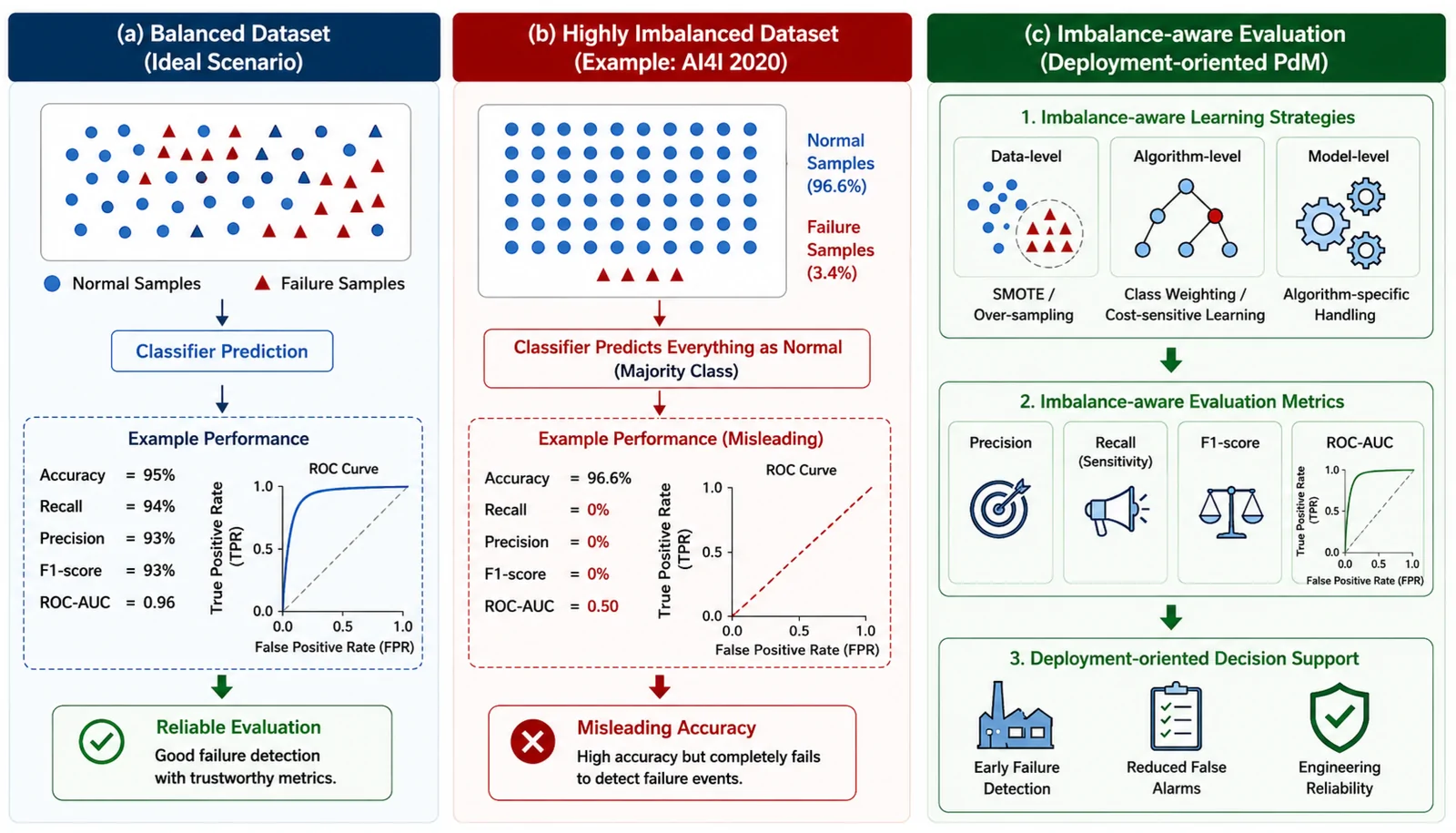
Conceptual illustration of imbalance-aware evaluation for deployment-oriented PdM. (**a**) When datasets are balanced, evaluation is dependable because normal and failure instances are represented in comparable proportions. (**b**) In strongly imbalanced industrial datasets, such as the AI4I 2020 dataset, overall classification accuracy can be deceptive, as predictions are dominated by the majority class. (**c**) Therefore, deployment-oriented PdM integrates imbalance-aware learning methods with evaluation metrics that emphasize the minority class, such as Precision, Recall, F1-score, and ROC-AUC, to achieve more trustworthy failure detection and better-informed engineering decisions.

As illustrated in Figure 3, aggregate classification accuracy alone is insufficient to adequately characterize PdM performance under pronounced class imbalance in operating conditions. Consequently, recent research increasingly adopts evaluation metrics that emphasize the minority class—such as Precision, Recall, F1-score, and ROC-AUC—in conjunction with imbalance-aware learning strategies, in order to obtain more reliable and informative assessments of predictive performance in realistic industrial environments.

While contemporary studies have significantly advanced PdM by improving learning algorithms and developing imbalance-aware approaches, model performance is still heavily shaped by how features are represented and how models are evaluated. Much of the existing work focuses on refining algorithms, whereas comparatively little effort has been directed toward combining engineering-informed feature design with standardized benchmark evaluations carried out under consistent experimental conditions. As a result, creating a unified framework that integrates engineered features, imbalance-aware learning, and deployment-oriented benchmarking remains a key research avenue for industrial PdM [42].

### 2.4. Deployment-Oriented Benchmarking and Evaluation

Benchmarking plays a fundamental role in PdM because objective comparison among machine learning algorithms requires standardized experimental protocols. Although numerous PdM studies report promising classification performance, direct comparison across different algorithms remains challenging because experimental settings often vary substantially. Differences in datasets, preprocessing procedures, feature representations, class-imbalance handling strategies, and evaluation metrics may all influence predictive performance, making it difficult to determine whether observed improvements originate from the learning algorithms themselves or from differences in experimental design [43,44].

The absence of standardized evaluation protocols is especially challenging in industrial PdM, where equipment failures occur only rarely. Under such highly imbalanced conditions, relying solely on conventional accuracy can be misleading, as a classifier that labels almost all instances as normal may still obtain a high overall accuracy while completely missing critical failure cases. As a result, metrics that are sensitive to the minority class, such as Precision, Recall, F1-score, and ROC-AUC, have become the preferred evaluation measures for PdM tasks [45,46].

In addition to predictive capability, real-world deployment requires PdM models to satisfy additional engineering requirements. Computational efficiency is essential for real-time monitoring in IIoT and edge-computing environments where processing resources are limited. Furthermore, engineering interpretability improves user confidence by enabling maintenance personnel to understand the physical basis of model predictions. Robustness under varying operating conditions is also essential because industrial equipment is exposed to diverse production environments, workloads, operating regimes, and sensor conditions, all of which may influence model reliability after deployment. Accordingly, deployment-oriented benchmarking should simultaneously evaluate predictive performance, computational efficiency, robustness, and engineering interpretability rather than relying solely on conventional classification accuracy.

While deployment-oriented benchmarking has attracted growing interest in recent PdM research, relatively few studies simultaneously integrate engineering-guided feature construction, standardized feature representations, imbalance-aware learning, and consistent evaluation protocols within a single experimental framework. Consequently, objective comparison among different machine learning paradigms remains challenging, and the reproducibility of reported results is often constrained. These limitations motivate the development of a deployment-oriented benchmarking methodology that controls the major components of the experimental pipeline under consistent conditions. As a result, it remains challenging to objectively compare different machine learning paradigms, and the reproducibility of experiments is frequently constrained. These issues provide the primary motivation for the deployment-oriented benchmarking framework developed in this study, which is presented in the next section.

The preceding literature review indicates that recent advances in predictive maintenance have primarily focused on improving individual aspects of the analytical pipeline, including feature engineering, machine learning algorithms, and deployment-oriented evaluation. However, these components are generally investigated independently, making objective comparison across different studies difficult due to variations in feature representations, preprocessing procedures, benchmark protocols, and evaluation criteria. To better illustrate the methodological positioning of the proposed framework, Table 2 compares representative PdM studies according to four key methodological components: physics-informed feature engineering, feature relevance analysis, benchmark protocols, and deployment-oriented evaluation. As shown, the proposed framework distinguishes itself by integrating these complementary components into a unified benchmarking methodology. These four methodological components correspond directly to the major design dimensions identified in Section 2.2, Section 2.3 and Section 2.4 and constitute the basis of the proposed Physics-Informed Benchmarking Framework.

### 2.5. Literature Summary and Research Motivation

The preceding literature review highlights that recent advances in IIoT technologies, physics-informed feature engineering, machine learning algorithms, and deployment-oriented evaluation have significantly improved the capability of predictive maintenance systems. Existing studies have demonstrated that each of these research directions contributes to more reliable fault diagnosis and equipment health assessment. Consequently, contemporary PdM has evolved from conventional fault prediction toward an integrated intelligent maintenance paradigm that combines sensing, data analytics, and engineering knowledge.

Despite these advances, the literature also reveals that these research directions have largely progressed independently. Many studies focus primarily on improving individual components of the PdM pipeline, such as feature engineering, learning algorithms, feature selection, or imbalance-aware learning, while relatively few investigate these components under a unified experimental framework. Furthermore, differences in feature representations, preprocessing procedures, evaluation metrics, and benchmark protocols make objective comparison among existing approaches difficult and often reduce the reproducibility of reported results.

The reviewed literature therefore suggests that future deployment-oriented PdM research should not only pursue higher predictive performance but also establish standardized and reproducible evaluation methodologies that enable fair comparison across representative learning paradigms while preserving engineering interpretability and practical applicability.

Motivated by these observations, this study develops a Physics-Informed Benchmarking Framework that integrates engineering-guided feature construction, feature relevance validation, standardized benchmark model development, imbalance-aware learning, and deployment-oriented evaluation into a unified experimental workflow. The following section presents the proposed methodology, including dataset preparation, physics-informed feature engineering, benchmark model development, and deployment-oriented evaluation.

## 3. Proposed Methodology

This section presents the complete research methodology adopted to develop and evaluate the proposed Physics-Informed Benchmarking Framework for deployment-oriented PdM. Rather than focusing solely on machine learning model development, the proposed methodology integrates physics-informed feature engineering, feature relevance analysis, imbalance-aware learning, and deployment-oriented performance evaluation into a unified experimental workflow. The methodology is designed to ensure reproducibility, fair benchmarking, and engineering interpretability throughout the entire PdM process.

### 3.1. Overall Physics-Informed Benchmarking Framework

This study proposes a Physics-Informed Benchmarking Framework that establishes a unified and reproducible methodology for deployment-oriented PdM. Rather than treating feature engineering, feature relevance analysis, benchmark model development, and performance evaluation as independent tasks, the proposed framework integrates these components into a coherent analytical workflow. This unified design ensures that all benchmark models are developed under identical experimental conditions while preserving engineering interpretability, experimental reproducibility, and practical deployment applicability. Consequently, the proposed methodology enables objective comparison among commonly adopted machine learning paradigms while explicitly incorporating engineering knowledge into the PdM process.

**Figure 4:**
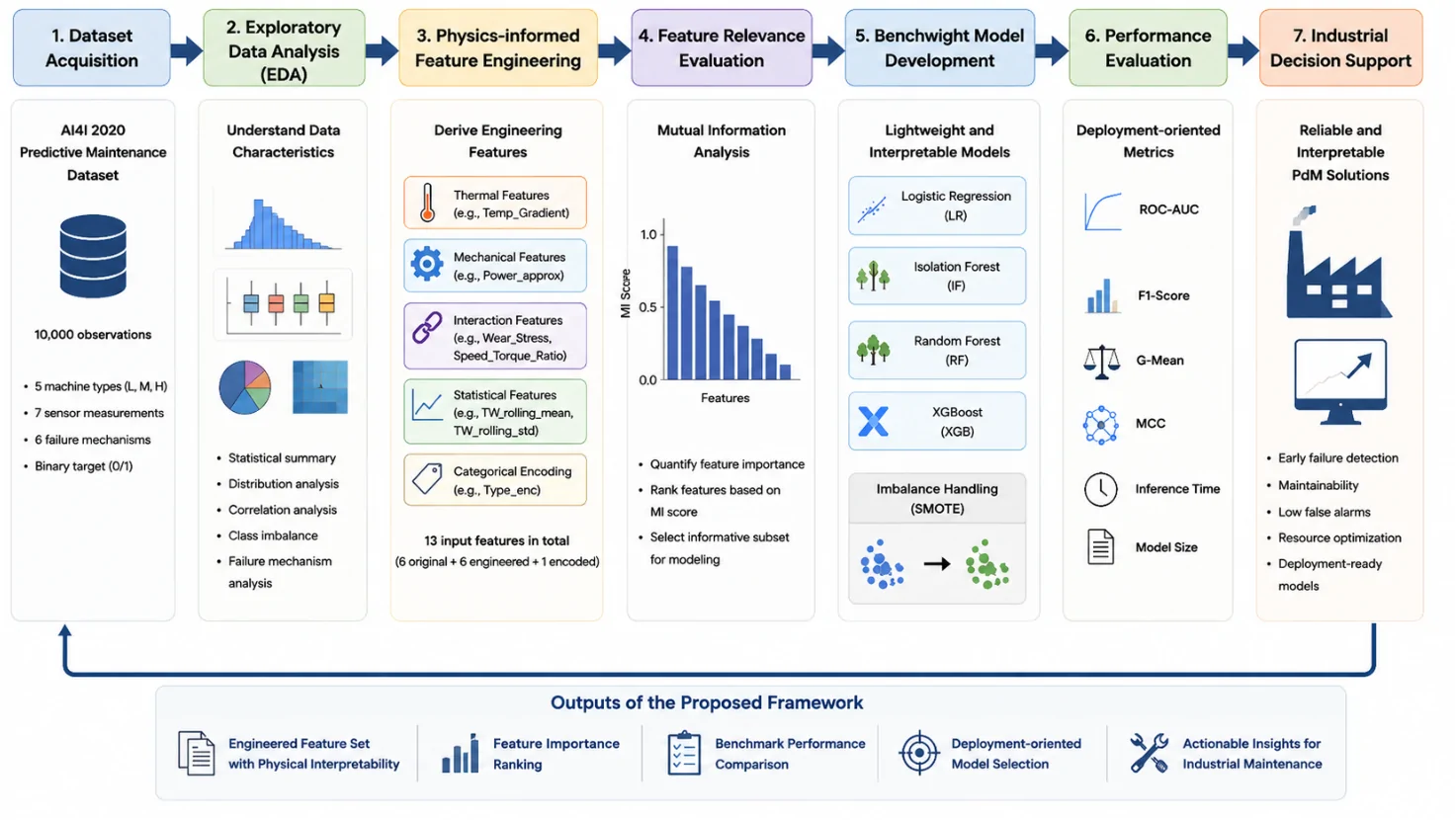
Overview of the proposed Physics-Informed Benchmarking Framework. The framework integrates experimental dataset preparation, physics-informed feature engineering, Mutual Information-based feature validation, benchmark model development, and deployment-oriented performance evaluation into a unified and reproducible workflow for industrial PdM.

Figure 4 provides an overview of the proposed Physics-Informed Benchmarking Framework. The framework organizes the entire PdM pipeline into five sequential stages, including dataset preparation, physics-informed feature engineering, feature relevance validation, benchmark model development, and deployment-oriented performance evaluation. Each component is described in detail in the following subsections.

The proposed framework establishes a standardized experimental pipeline that integrates engineering knowledge with data-driven learning while ensuring reproducibility and fair comparison among widely used ML paradigms. The following sections describe each component of the framework in detail.

The first three stages establish the data and feature foundation of the proposed framework. The AI4I 2020 Predictive Maintenance Dataset serves as the standardized experimental benchmark for model development. EDA is first conducted to investigate statistical characteristics and identify physically meaningful relationships among multisource sensor variables. Based on these observations, engineering knowledge is incorporated to construct physics-informed features that better characterize thermal behavior, mechanical loading, degradation progression, and operating conditions than the original sensor measurements alone.

The subsequent stages establish the unified benchmark model development protocol. Mutual Information is employed to evaluate feature relevance and identify informative variables before representative machine learning algorithms are developed under identical preprocessing, imbalance-handling, and training procedures. Logistic Regression, Isolation Forest, Random Forest, and XGBoost are selected because they represent complementary learning paradigms that are widely adopted in industrial PdM. Finally, deployment-oriented performance evaluation assesses predictive capability, computational efficiency, robustness under severe class imbalance, and engineering interpretability. Collectively, these stages transform validated analytical results into practical maintenance knowledge that supports industrial decision-making.

The proposed methodology differs from conventional PdM studies in three important aspects. First, engineering knowledge is explicitly incorporated during feature construction instead of relying solely on raw sensor measurements. Second, feature relevance is systematically validated before benchmark model development, thereby reducing feature redundancy while preserving engineering interpretability. Third, all benchmark algorithms are trained and evaluated under an identical experimental protocol, ensuring fair comparison, experimental reproducibility, and deployment-oriented evaluation. These three principles collectively constitute the core design philosophy of the proposed Physics-Informed Benchmarking Framework.

The following sections describe each stage of the proposed methodology in detail. Section 3.2 introduces the experimental dataset, followed by the physics-informed feature engineering methodology, feature relevance analysis using Mutual Information, benchmark model development, and deployment-oriented performance evaluation. Together, these components establish a unified and reproducible framework for industrial PdM.

### 3.2. Experimental Dataset

The AI4I 2020 Predictive Maintenance Dataset was adopted as the benchmark dataset for developing and validating the proposed Physics-Informed Benchmarking Framework. The dataset has become one of the most widely used public benchmarks for PdM research because it provides realistic industrial operating conditions, multiple failure mechanisms, and multisource IIoT sensor measurements. Compared with proprietary industrial datasets, which are often inaccessible due to confidentiality restrictions and limited public availability, the AI4I dataset facilitates experimental reproducibility, enables objective comparison with existing studies, and provides a standardized benchmark for evaluating the proposed Physics-Informed Benchmarking Framework.

Among publicly available predictive maintenance datasets, the AI4I 2020 dataset was selected because it simultaneously provides multiple physical failure mechanisms, multisource sensor measurements, machine descriptors, and a severely imbalanced failure distribution within a standardized benchmark. These characteristics closely match the objectives of this study, which emphasize physics-informed feature engineering, fair benchmark comparison, and deployment-oriented evaluation under realistic industrial operating conditions. Consequently, the AI4I dataset offers a more appropriate benchmark than many publicly available datasets that focus on a single failure mode, limited sensor variables, or application-specific operating environments.

The dataset contains 10,000 operating records collected from a simulated machining process and includes five continuous sensor variables, one categorical machine descriptor, and a binary failure label. The numerical variables comprise air temperature, process temperature, rotational speed, torque, and tool wear, while the machine descriptor represents three production quality levels (L, M, and H). In addition, each observation is associated with one of five physical failure mechanisms: tool wear failure (TWF), heat dissipation failure (HDF), power failure (PWF), overstrain failure (OSF), and random failure (RNF). For benchmark classification, these failure mechanisms are aggregated into a binary target representing normal and failure conditions.

One of the most important characteristics of the AI4I dataset is its highly imbalanced class distribution. Failure observations account for approximately 3.4% of the entire dataset, corresponding to an imbalance ratio of approximately 28:1 between normal and failure samples. Such severe class imbalance reflects practical industrial environments in which equipment failures occur infrequently during routine production but remain critically important for maintenance decision-making. Consequently, the AI4I dataset provides a realistic benchmark for evaluating the robustness of PdM models under practical industrial operating conditions.

**Figure 5:**
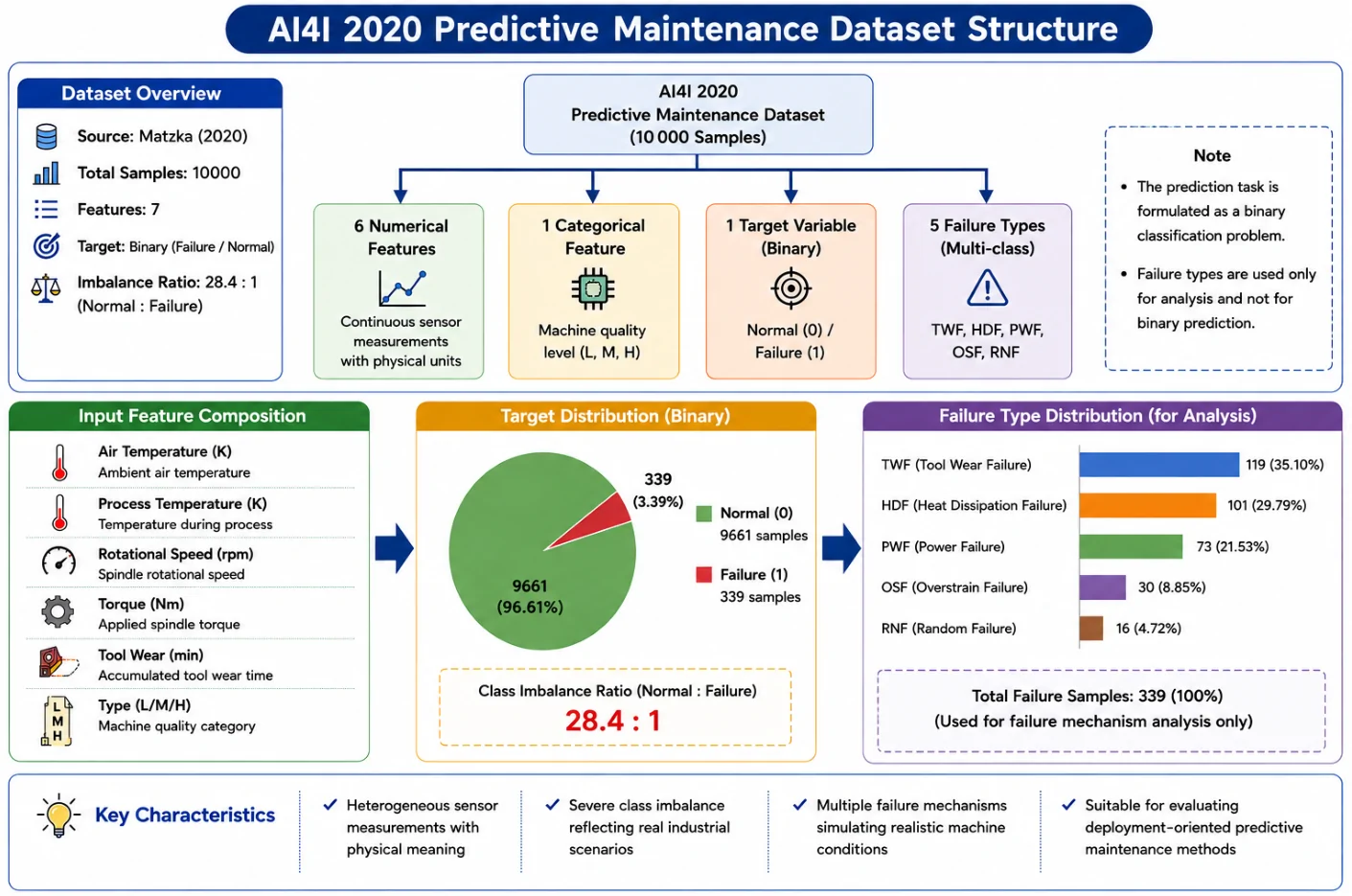
Overview of the AI4I 2020 Predictive Maintenance Dataset. The dataset comprises multisource IIoT sensor measurements, machine descriptors, and binary failure labels representing realistic industrial operating conditions. Multiple physical failure mechanisms together with severe class imbalance make the dataset suitable for evaluating deployment-oriented PdM methods.

Figure 5 provides an overview of the AI4I 2020 Predictive Maintenance Dataset adopted in this study. The dataset contains industrial sensor measurements describing machine operating conditions together with machine descriptors and failure labels. These characteristics establish the data foundation for the subsequent exploratory data analysis and physics-informed feature engineering procedure.

As illustrated in Figure 5, the AI4I dataset combines multi-sensor variables with multiple physical failure mechanisms under a highly imbalanced class distribution. These characteristics closely resemble practical industrial environments and therefore provide an appropriate benchmark for evaluating the proposed Physics-Informed Benchmarking Framework.

Table 3 describes the variables contained in the AI4I dataset together with their physical meanings and corresponding roles in the proposed framework. These variables provide the basis for the subsequent construction of physics-informed features.

The AI4I dataset offers a comprehensive representation of machine operating conditions through multisource thermal, mechanical, degradation-related, and categorical variables. These variables establish the analytical foundation for the subsequent exploratory data analysis, where statistically and physically meaningful relationships among the original sensor measurements are investigated to support the construction of physics-informed features.

### 3.3. Physics-Informed Feature Engineering

Physics-informed feature engineering constitutes the core component of the proposed benchmarking framework. Rather than relying solely on the original IIoT sensor measurements, this study incorporates engineering knowledge to construct physically meaningful variables that better characterize thermal behavior, mechanical loading, degradation progression, and operational efficiency. Unlike purely data-driven feature construction, the proposed methodology explicitly exploits physical relationships among heterogeneous sensor variables to improve both predictive capability and engineering interpretability.

**Figure 6:**
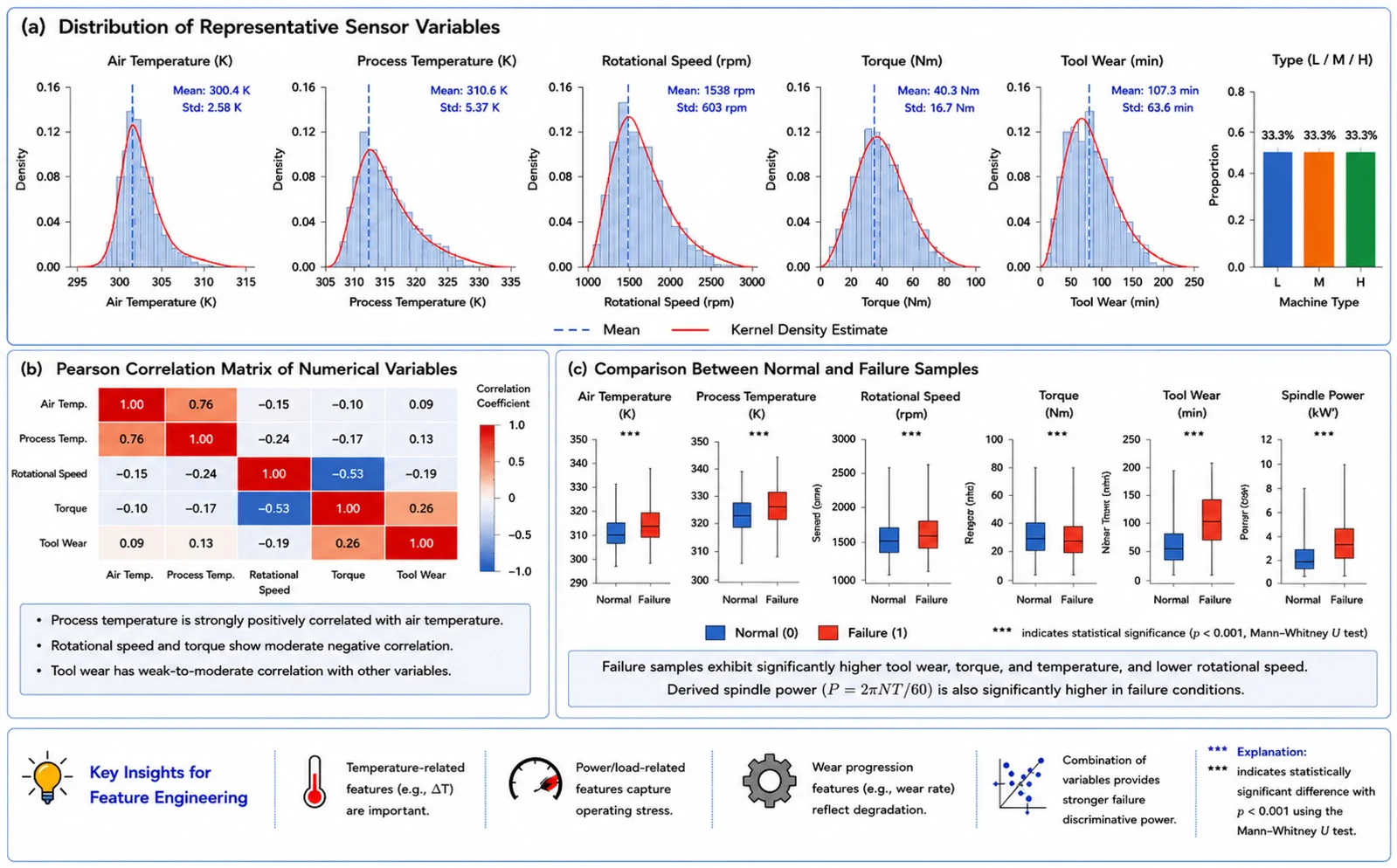
Exploratory data analysis of the AI4I 2020 Predictive Maintenance Dataset. The multisource distributions and relationships among the original sensor variables reveal distinct thermal and mechanical operating characteristics, providing the engineering motivation for constructing physics-informed derived features in the subsequent feature engineering stage.

Figure 6 presents the exploratory data analysis performed on the AI4I 2020 Predictive Maintenance Dataset. The objective of this analysis is not only to examine the statistical characteristics of the original sensor variables but also to identify physically meaningful relationships that can guide the construction of engineering-derived features.

As illustrated in Figure 6, the original sensor variables exhibit distinct thermal and mechanical characteristics together with observable interactions among operating conditions. These observations indicate that meaningful physical relationships exist beyond the original measurements and therefore motivate the construction of physics-informed engineered features described in the following section.

**Figure 7:**
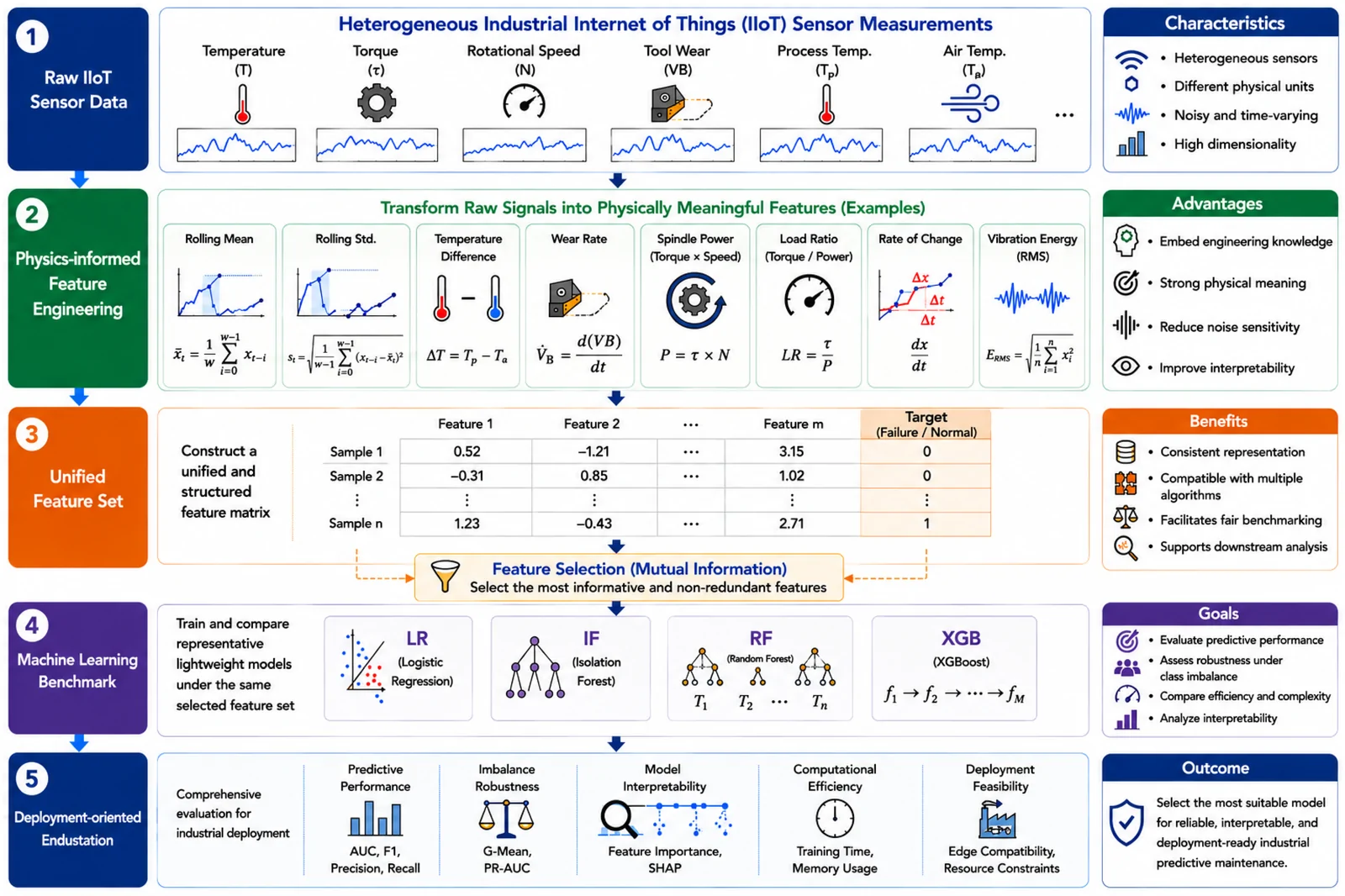
Workflow of the proposed physics-informed feature engineering methodology. Guided by exploratory data analysis and engineering knowledge, the original IIoT sensor variables are transformed into physically meaningful engineered features through thermal, mechanical, interaction, and statistical feature construction. The resulting feature representation provides the foundation for subsequent feature relevance validation.

Figure 7 illustrates the workflow of the proposed physics-informed feature engineering methodology. Guided by the exploratory observations presented in Section 3.3, engineering knowledge is incorporated into the original IIoT sensor variables to construct physically meaningful engineered features that better characterize thermal behavior, mechanical loading, degradation progression, and operational efficiency.

As illustrated in Figure 7, the proposed methodology organizes feature construction into four complementary categories: thermal, mechanical, interaction, and statistical features. Rather than introducing arbitrary mathematical transformations, each engineered feature is designed according to explicit engineering principles, thereby enriching the original feature representation while preserving physical interpretability. The validated feature representation is subsequently evaluated using Mutual Information, as described in the following section.

Following the workflow shown in Figure 7, seven engineered variables were derived from the original AI4I sensor readings. Each feature was constructed based on engineering principles informed by the earlier exploratory analysis, rather than on arbitrary mathematical manipulations. The mathematical definitions of each feature category are detailed below.

#### 3.3.1. Thermal Feature

To characterize thermal loading during machining, the temperature gradient is defined as

(1) $$\Delta T=T_{p}-T_{a},$$

where $T_{p}$ and $T_{a}$ denote the process temperature and air temperature, respectively. The temperature gradient represents the thermal difference between the machining process and the surrounding environment and serves as an indicator of heat dissipation conditions. Since excessive thermal accumulation is closely associated with heat dissipation failure (HDF), this feature provides a physically meaningful descriptor for thermal degradation.

#### 3.3.2. Mechanical Feature

Mechanical loading was characterized using the approximate spindle power computed from rotational speed and torque,

(2) $$P_{\mathrm{approx}}=\frac{\tau N}{9550},$$

where τ denotes torque (Nm), *N* denotes rotational speed (rpm), and the constant 9550 converts the torque–speed product into approximate spindle power expressed in kilowatts according to the conventional rotating machinery power relationship. This feature summarizes the mechanical workload experienced by the spindle and provides a more physically interpretable representation than either torque or rotational speed alone.

#### 3.3.3. Interaction Features

Machine degradation is generally governed by the interaction between multiple physical variables rather than isolated sensor measurements. Therefore, two interaction-based features were constructed.

The wear–stress index combines accumulated tool wear and spindle torque,

(3) $$S_{\mathrm{wear}}=W\tau ,$$

where *W* denotes tool wear and τ denotes torque. This feature represents the combined influence of progressive tool degradation and mechanical loading, thereby emphasizing operating conditions in which worn tools are subjected to high torque demand.

The second interaction feature is the speed–torque ratio,

(4) $$R_{\mathrm{ST}}=\frac{N}{\tau +\epsilon },$$

where ϵ denotes a small constant introduced to avoid numerical instability when torque approaches zero. This ratio characterizes operational efficiency by relating spindle rotational output to the applied mechanical load, thereby providing complementary information beyond the original sensor measurements.

#### 3.3.4. Statistical Features

To approximate local degradation behavior, rolling statistics were calculated after ordering observations according to the sample identifier. Although the AI4I dataset does not explicitly provide temporal sequences, neighboring observations represent consecutive production records and therefore provide an approximate description of local degradation trends.

The rolling mean of tool wear is defined as

(5) $${\bar{W}}_{t}^{\left(w\right)}=\frac{1}{w}\sum_{i=t-w+1}^{t}W_{i},$$

where w=50 denotes the rolling window length.

The rolling standard deviation is computed as

(6) $$\sigma_{W,t}^{\left(w\right)}=\sqrt{\frac{1}{w-1}\sum_{i=t-w+1}^{t}{\left(W_{i}-{\bar{W}}_{t}^{\left(w\right)}\right)}^{2}},$$

where w=50. Together, these two statistical descriptors characterize local degradation tendency and degradation variability, providing complementary information regarding wear evolution.

#### 3.3.5. Categorical Feature

The categorical machine type was encoded into numerical form to facilitate machine learning implementation,

(7) $$T_{\mathrm{enc}}=\left\{\begin{array}{cc} 0, & \mathrm{Type}\;=\;L,\\ 1, & \mathrm{Type}\;=\;M,\\ 2, & \mathrm{Type}\;=\;H. \end{array}\right.$$

This encoding preserves the ordinal production quality information provided by the AI4I dataset while enabling machine type to participate in subsequent feature relevance analysis and benchmark model development.

Table 4 summarizes the engineering-derived variables together with their corresponding physical interpretations and expected contributions to PdM. Each feature is designed according to explicit engineering principles rather than arbitrary mathematical transformation, thereby providing interpretable representations of machine operating conditions.

As outlined in Table 4, the proposed feature engineering approach augments the raw sensor data by integrating thermal, mechanical, interaction-based, statistical, and production-related aspects into a single, unified feature space. Relative to the original AI4I measurements, these engineered variables offer more informative characterizations of machine degradation while maintaining clear physical interpretability. This enriched feature set is then assessed using Mutual Information to determine the relevance of both the original and engineered variables prior to constructing the benchmark models.

Taken together, these exploratory findings suggest that the raw IIoT sensor signals possess significant underlying physical dependencies that can be leveraged to create more informative feature representations. Instead of depending only on individual sensor readings, the proposed approach deliberately embeds these physical relationships into the engineered feature design. The resulting physics-guided feature engineering pipeline is illustrated in Figure 7.

### 3.4. Feature Relevance Analysis Using Mutual Information

Following the construction of the physics-informed engineered features, feature relevance analysis was performed to objectively evaluate the predictive contribution of both the original sensor variables and the engineered features before benchmark model development. Although the engineered features were designed according to explicit engineering principles, their predictive usefulness should be quantitatively validated rather than assumed. Therefore, feature relevance analysis was incorporated into the proposed Physics-Informed Benchmarking Framework to establish a standardized feature representation for all benchmark ML models.

Mutual Information (MI) was adopted because it quantifies the statistical dependency between an input feature and the target variable without assuming linear relationships. Unlike conventional correlation coefficients, MI effectively captures both linear and nonlinear associations and is therefore particularly suitable for multisource IIoT sensor variables. Furthermore, MI is a model-independent criterion, allowing feature relevance to be evaluated prior to classifier training while avoiding algorithm-specific bias. Although alternative methods such as SHAP, Permutation Importance, and ReliefF are also widely adopted for feature relevance analysis, SHAP and Permutation Importance generally require a trained predictive model and therefore depend on the selected learning algorithm, whereas ReliefF primarily evaluates feature discrimination based on instance similarity. In contrast, MI provides a unified, model-independent criterion that enables consistent feature validation before benchmark model development, which is more closely aligned with the objective of establishing a standardized benchmarking framework. Consequently, MI provides an objective and reproducible basis for feature validation within the proposed benchmark framework.

Alternative feature relevance techniques, such as SHAP, Permutation Importance, and ReliefF, were also considered during the methodological design. However, SHAP and Permutation Importance require a trained prediction model and therefore produce model-dependent feature importance, while ReliefF relies on neighborhood-based distance estimation and may be sensitive to data distribution and class imbalance. In contrast, Mutual Information evaluates feature relevance independently of any specific learning algorithm, enabling the proposed physics-informed feature engineering strategy to be validated objectively before benchmark model development. This model-independent characteristic is consistent with the objective of establishing a fair and reproducible benchmarking framework.

For a feature variable *X* and target variable *Y*, the Mutual Information is defined as

(8) $$I\left(X;Y\right)=\sum_{x\in X}\sum_{y\in Y}p\left(x,y\right)\log\left(\frac{p(x,y)}{p(x)p(y)}\right),$$

where p(x) and p(y) denote the marginal probability distributions of the feature and target variables, respectively, while p(x,y) represents their joint probability distribution. A larger MI value indicates that the corresponding feature contains greater information regarding the target variable and is therefore expected to contribute more substantially to PdM performance. Conversely, features with relatively low MI values provide limited additional predictive information and may be excluded from subsequent benchmark model development.

**Figure 8:**
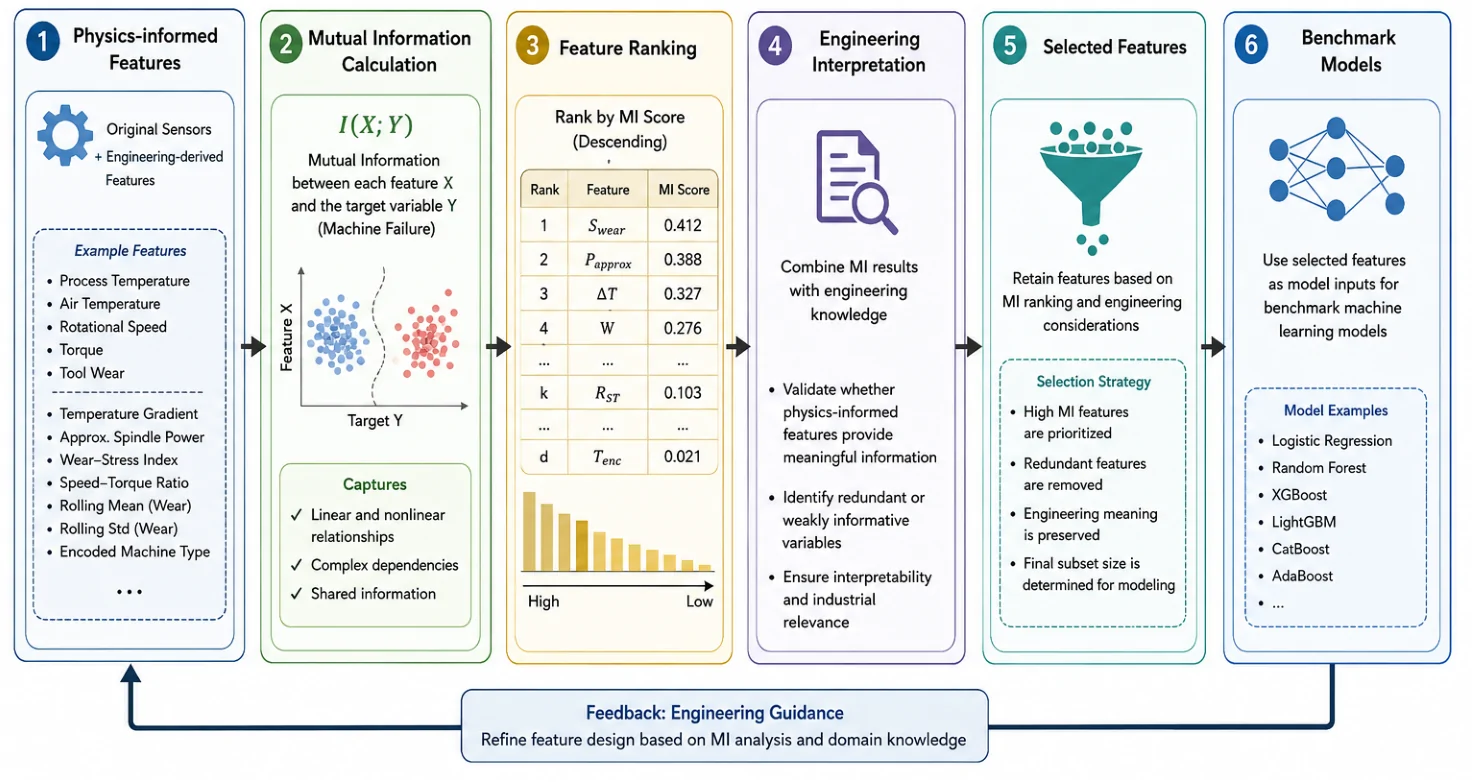
Workflow of the Mutual Information-based feature relevance analysis. Both the original sensor variables and the physics-informed engineered features are evaluated using a unified Mutual Information criterion to quantify their predictive relevance. The validated feature representation subsequently serves as the common input for benchmark model development.

Figure 8 illustrates the workflow of the Mutual Information-based feature relevance analysis adopted in the proposed Physics-Informed Benchmarking Framework. Following physics-informed feature engineering, both the original sensor variables and the engineered features are evaluated using a unified relevance criterion to quantify their predictive contribution before benchmark model development.

As illustrated in Figure 8, feature relevance analysis constitutes the intermediate validation stage between feature engineering and benchmark model development. Rather than relying solely on statistical ranking for feature selection, the proposed framework combines Mutual Information analysis with engineering interpretation to validate whether the engineered features effectively represent physically meaningful degradation characteristics. The resulting validated feature representation is subsequently employed for standardized benchmark model development.

Table 5 summarizes the Mutual Information-based feature relevance analysis protocol adopted in the proposed framework. The protocol standardizes the feature validation procedure by defining the evaluation method, feature set, target variable, selection principle, and analysis output before benchmark model development.

Unlike conventional feature selection methods that rely solely on statistical ranking, the proposed framework combines Mutual Information analysis with engineering interpretation. Accordingly, the objective of feature relevance analysis is not merely to maximize dimensionality reduction but to validate whether the engineered features successfully capture physically meaningful degradation characteristics. Features exhibiting moderate MI values may therefore be retained when they represent important physical mechanisms or provide valuable engineering insight for industrial maintenance decision-making. This strategy preserves both predictive capability and engineering interpretability within the validated feature space.

The validated feature representation established through MI analysis provides the standardized input feature space for all benchmark ML models. Quantitative MI scores and feature rankings are presented and discussed in Section 4.2, whereas the following section introduces the representative benchmark ML algorithms developed under an identical preprocessing, training, and evaluation protocol.

### 3.5. Benchmark Model Development

After validating feature relevance, representative benchmark ML models were constructed under a unified experimental protocol. Instead of seeking a single universally superior prediction algorithm, the proposed Physics-Informed Benchmarking Framework is designed to provide a standardized benchmarking setting in which widely used learning paradigms are fairly compared using an identical, validated feature representation, consistent preprocessing steps, uniform handling of class imbalance, and a common evaluation procedure. As a result, performance differences observed in the benchmark primarily stem from the intrinsic learning properties of the algorithms, rather than from variations in experimental design.

To provide comprehensive benchmark coverage, four typical ML algorithms were selected: Logistic Regression (LR), Isolation Forest (IF), Random Forest (RF), and XGBoost. Collectively, these algorithms represent linear supervised learning, unsupervised anomaly detection, bagging-based ensemble learning, and boosting-based ensemble learning, respectively. This selection enables systematic comparison across diverse learning paradigms commonly adopted in industrial PdM while maintaining practical relevance for deployment-oriented applications.

**Figure 9:**
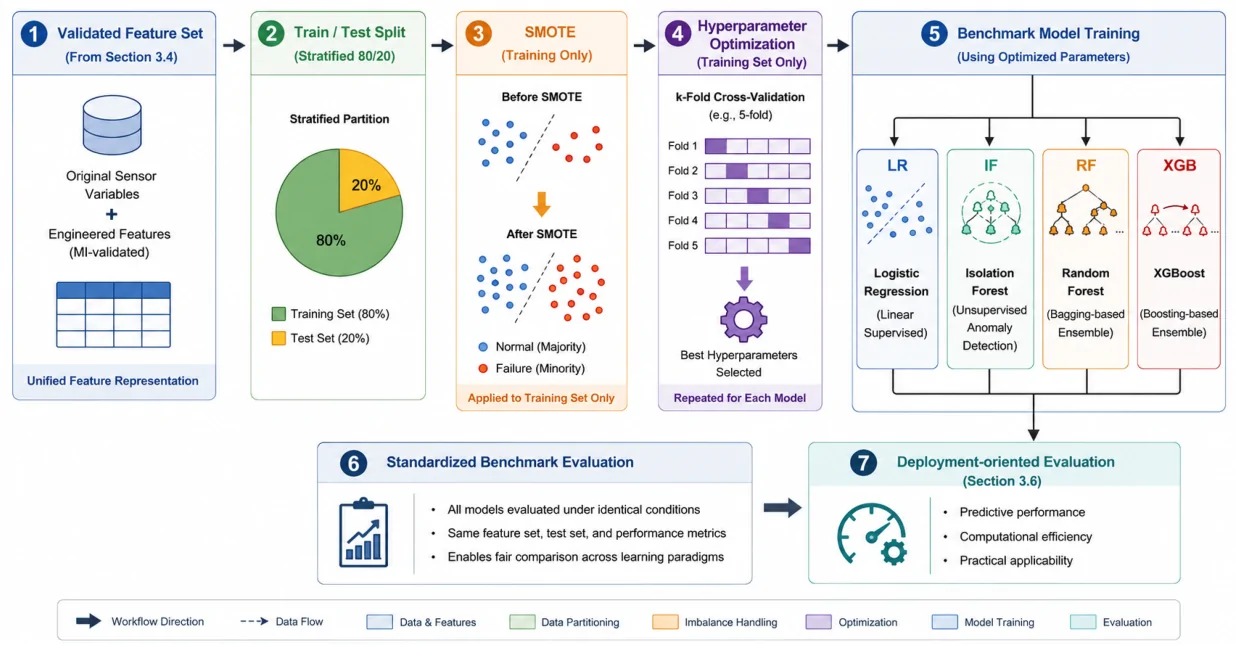
Unified workflow for benchmark model development. Following feature relevance validation, all benchmark ML models are developed under identical train–test partitioning, preprocessing, class imbalance treatment, and hyperparameter configuration procedures. This standardized workflow enables objective, reproducible, and deployment-oriented comparison among representative learning paradigms.

Figure 9 illustrates the workflow of benchmark model development within the proposed Physics-Informed Benchmarking Framework. Using the validated feature representation established in Section 3.4, all benchmark ML models are developed under a unified experimental protocol that includes identical train–test partitioning, preprocessing procedures, class imbalance treatment, and hyperparameter configuration. This standardized workflow establishes a fair and reproducible benchmarking environment for industrial PdM.

As illustrated in Figure 9, the proposed framework standardizes every stage of benchmark model development while preserving the intrinsic learning characteristics of individual algorithms. Consequently, observed differences in predictive performance primarily reflect differences among learning paradigms rather than inconsistencies in feature representation, preprocessing procedures, or class imbalance handling.

To ensure a fair and reproducible benchmark comparison, representative hyperparameter settings were manually specified according to widely adopted practices for each benchmark algorithm and then applied consistently throughout all experiments. The objective of this study is not to maximize the performance of individual classifiers through extensive hyperparameter optimization, but rather to establish a standardized benchmarking framework under identical experimental conditions. Therefore, all benchmark models employed fixed hyperparameter settings together with identical data partitioning, feature representations, preprocessing procedures, class-imbalance handling strategies, and evaluation metrics to enable objective performance comparison.

Table 6 summarizes the representative benchmark ML models together with their principal hyperparameter configurations and corresponding roles within the proposed framework. Rather than optimizing each algorithm independently, parameter settings commonly adopted in previous studies were employed to maintain a consistent, reproducible, and deployment-oriented benchmarking environment.

As outlined in Table 6, we adopted commonly used hyperparameter settings reported in prior studies rather than performing exhaustive model-specific optimization. This design choice is consistent with the objective of the proposed Physics-Informed Benchmarking Framework, which emphasizes standardized and reproducible comparison across representative learning paradigms instead of maximizing the performance of individual algorithms. Accordingly, the selected hyperparameter values can be regarded as representative baseline configurations that facilitate fair benchmarking under a unified experimental protocol.

#### 3.5.1. Logistic Regression

Logistic Regression (LR) was selected as the baseline supervised classifier because of its computational efficiency, high interpretability, and widespread use in industrial classification problems. As a linear model, LR provides an important reference for evaluating whether the proposed physics-informed engineered features improve class separability without relying on complex nonlinear decision boundaries.

The probability of machine failure is estimated as

(9) $$P\left(y=1|x\right)=\frac{1}{1+\exp[-\left(\beta_{0}+\beta^{T}x\right)]},$$

where x denotes the input feature vector, β represents the regression coefficients, and $\beta_{0}$ is the intercept term. Although LR assumes a linear relationship between the feature space and the decision boundary, it serves as an interpretable baseline for assessing the effectiveness of the validated physics-informed feature representation.

#### 3.5.2. Isolation Forest

Isolation Forest (IF) was included as the widely used unsupervised anomaly detection algorithm within the benchmark framework. Unlike supervised classifiers, IF isolates anomalous observations through random recursive partitioning without requiring failure labels during model construction. Samples exhibiting shorter average path lengths are more likely to be identified as anomalies.

This learning paradigm is particularly suitable for industrial PdM applications, where failure observations are typically scarce and highly imbalanced. Including IF enables objective comparison between supervised classification and unsupervised anomaly detection under the same validated feature representation.

#### 3.5.3. Random Forest

Random Forest (RF) was selected as the typical bagging-based ensemble learning algorithm because of its robustness to noisy industrial data and its ability to model nonlinear feature interactions. Final predictions are obtained through majority voting among multiple decision trees,

(10) $$\hat{y}=\mathrm{mode}\left\{T_{1}\left(x\right),T_{2}\left(x\right),\ldots ,T_{K}\left(x\right)\right\},$$

where $T_{k}\left(x\right)$ denotes the prediction generated by the *k*-th decision tree. In addition to providing robust predictive performance, RF also offers intrinsic feature importance estimation, making it well suited for validating the proposed physics-informed feature representation in industrial PdM.

#### 3.5.4. Extreme Gradient Boosting

XGBoost was selected as the representative boosting-based ensemble learning algorithm owing to its strong predictive capability on structured industrial datasets. XGBoost sequentially constructs decision trees by minimizing a regularized objective function,

(11) $$L=\sum_{i=1}^{n}l\left(y_{i},{\hat{y}}_{i}\right)+\sum_{k}\Omega \left(f_{k}\right),$$

where l(·) denotes the training loss function and Ω(·) represents the regularization term controlling model complexity. Within the proposed benchmark framework, XGBoost serves as the high-performance supervised learning benchmark for evaluating the effectiveness of the validated physics-informed feature representation.

#### 3.5.5. Experimental Implementation

To ensure objective comparison among all benchmark ML models, a unified experimental protocol was adopted throughout this study. The validated feature representation established in Section 3.4 served as the common input for every learning algorithm. The dataset was partitioned into training and testing subsets using stratified sampling with an 80:20 ratio to preserve the original class distribution. Feature standardization was performed using statistics computed exclusively from the training subset, and the resulting transformation parameters were subsequently applied to the testing subset to eliminate information leakage.

Because machine failure represents the minority class, the SMOTE was applied only to the training subset after data partitioning, whereas the testing subset remained unchanged to provide an unbiased assessment of model generalization capability. Hyperparameter settings followed commonly adopted configurations reported in previous studies rather than extensive model-specific optimization, as summarized in Table 6. Consequently, all benchmark ML models were developed under identical feature representations, preprocessing procedures, class imbalance treatment, and training protocols, ensuring that subsequent performance differences primarily reflect the intrinsic characteristics of the learning algorithms.

The benchmark models and unified experimental protocol outlined above form the learning core of the proposed Physics-Informed Benchmarking Framework. By standardizing feature representation, preprocessing steps, class imbalance handling, and model training, the framework supports objective and reproducible comparisons across key ML paradigms. The next section introduces the deployment-focused evaluation methodology used for model assessment.

### 3.6. Deployment-Oriented Performance Evaluation

The benchmark ML models were evaluated using a deployment-oriented performance assessment protocol designed to reflect practical industrial PdM requirements. Because machine failure is a rare event, overall classification accuracy alone cannot adequately characterize model performance. A classifier that predicts every observation as the normal class may still achieve high accuracy while completely failing to detect machine failures. Consequently, multiple complementary evaluation metrics were adopted to assess predictive capability from different perspectives, including failure detection, false-alarm control, classification robustness, computational efficiency, and deployment applicability.

**Figure 10:**
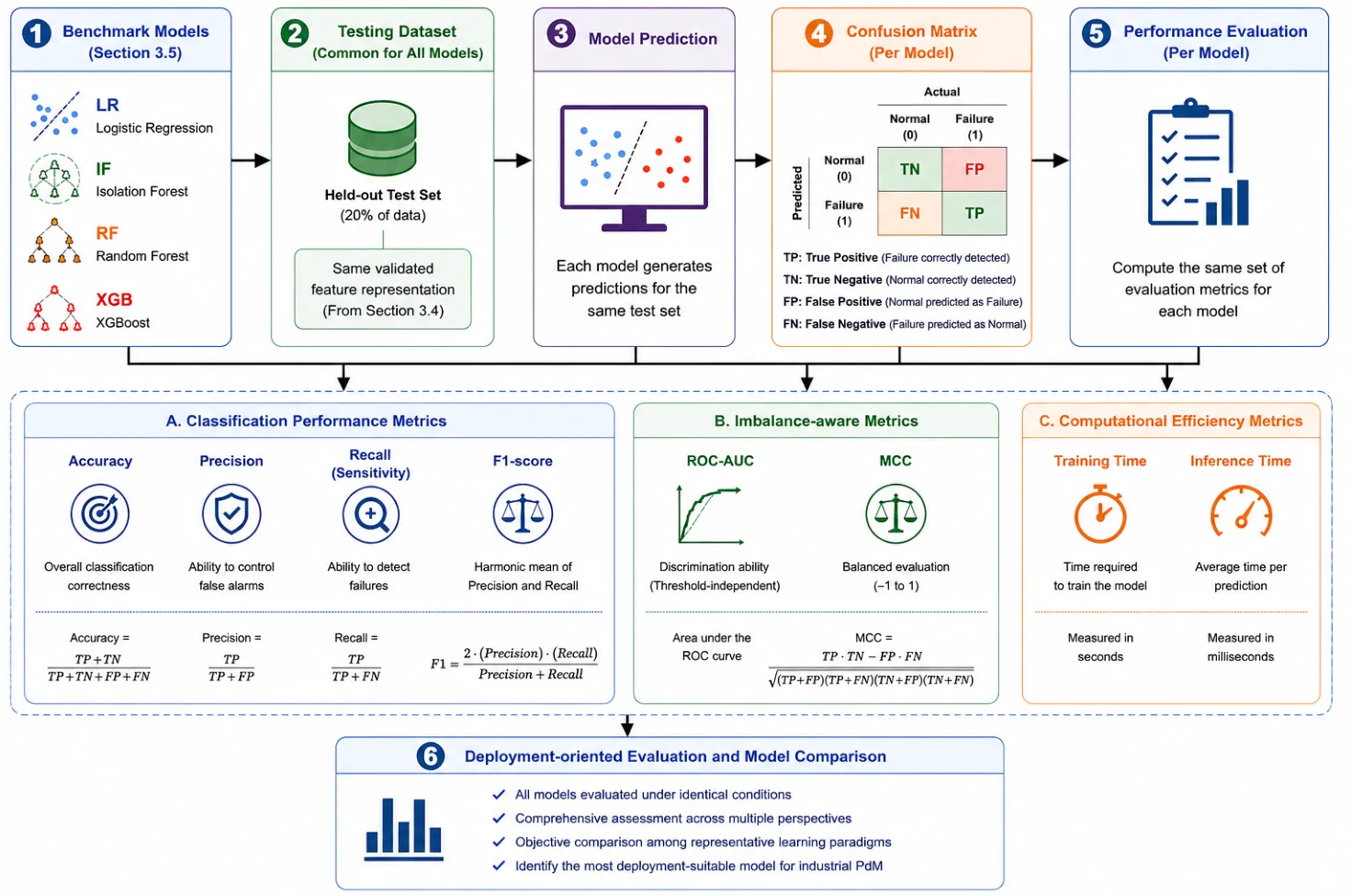
Workflow of the deployment-oriented performance evaluation. All benchmark ML models are evaluated using the identical testing dataset together with complementary performance metrics that quantify classification accuracy, class imbalance robustness, computational efficiency, and practical deployment capability. This standardized evaluation protocol provides an objective basis for benchmark comparison.

Figure 10 illustrates the workflow of the deployment-oriented performance evaluation adopted in the proposed Physics-Informed Benchmarking Framework. After benchmark model development, all commonly adopted ML models are evaluated using the identical testing dataset and a unified set of complementary performance metrics. This standardized evaluation protocol enables objective comparison of predictive capability, robustness under class imbalance, computational efficiency, and practical deployment applicability.

As illustrated in Figure 10, every benchmark model is assessed under identical evaluation conditions using the same testing dataset and performance metrics. Consequently, benchmark results reflect the predictive capability of different learning paradigms rather than variations in evaluation methodology. The deployment-oriented evaluation metrics adopted in this study are summarized in Table 7.

Unlike balanced classification problems, PdM datasets are typically dominated by healthy operating samples, while failure events constitute only a small proportion of the observations. Under such circumstances, overall classification accuracy alone may provide an overly optimistic assessment because a classifier predicting all samples as normal can still achieve high accuracy while completely failing to detect equipment failures. Therefore, deployment-oriented PdM requires multiple complementary performance indicators capable of evaluating minority-class detection, false-alarm control, and practical engineering applicability.

The overall classification accuracy is computed as

(12) $$Accuracy=\frac{\mathrm{TP}+\mathrm{TN}}{\mathrm{TP}+\mathrm{FP}+\mathrm{TN}+\mathrm{FN}},$$

where TP, TN, FP, and FN denote true positives, true negatives, false positives, and false negatives, respectively.

Prediction reliability is evaluated using Precision,

(13) $$Precision=\frac{\mathrm{TP}}{\mathrm{TP}+\mathrm{FP}},$$

which measures the proportion of predicted failures that correspond to actual failures. High precision indicates a low false-alarm rate and therefore reduces unnecessary maintenance interventions.

Recall (Sensitivity) is defined as

(14) $$Recall=\frac{\mathrm{TP}}{\mathrm{TP}+\mathrm{FN}},$$

which evaluates the ability of a model to correctly identify actual failure events. Since missed failures may result in unexpected equipment breakdowns, recall is regarded as one of the most critical metrics in PdM applications.

To balance Precision and Recall, the harmonic mean is computed as

(15) $$F_{1}=2\cdot \frac{Precision\times Recall}{Precision+Recall},$$

providing an overall indicator of minority-class prediction performance under imbalanced learning conditions.

Because of the severe class imbalance, Balanced Accuracy is additionally employed,

(16) $$Balanced\;Accuracy=\frac{Recall+Specificity}{2},$$

where

(17) $$Specificity=\frac{\mathrm{TN}}{\mathrm{TN}+\mathrm{FP}}.$$

Balanced Accuracy assigns equal importance to normal and failure classes and therefore provides a fairer assessment than conventional accuracy.

In addition to threshold-based performance measures, ROC-AUC and the Matthews Correlation Coefficient (MCC) are emphasized as the principal evaluation metrics in this study. ROC-AUC measures the ability of a classifier to discriminate between failure and normal samples across all possible decision thresholds, thereby reducing dependence on a specific classification threshold. Meanwhile, MCC simultaneously considers true positives, true negatives, false positives, and false negatives, providing a balanced and reliable assessment under highly imbalanced predictive maintenance conditions where failure samples constitute the minority class. The complementary use of ROC-AUC and MCC therefore enables a more comprehensive evaluation of discrimination capability and overall classification quality than relying solely on threshold-dependent metrics such as Accuracy or F1-score.

Table 7 summarizes the deployment-oriented evaluation metrics adopted in the proposed benchmark framework. Rather than relying solely on overall classification accuracy, complementary metrics are employed to assess failure detection capability, robustness under class imbalance, computational efficiency, and practical deployment applicability from multiple perspectives.

Each evaluation metric emphasizes a different aspect of predictive performance. Accuracy measures overall classification correctness, Precision reflects false-alarm control, Recall evaluates failure detection capability, F1-score balances Precision and Recall, ROC-AUC quantifies discrimination capability across classification thresholds, MCC provides a balanced overall assessment by jointly considering true positives, true negatives, false positives, and false negatives under severe class imbalance, while computational time reflects deployment efficiency. Together, these complementary metrics provide a comprehensive basis for benchmark comparison.

**Table 7:** Summary of deployment-oriented evaluation metrics adopted in the proposed Physics-Informed Benchmarking Framework.

Metric	Equation	Engineering Meaning	Role in Deployment
Accuracy	Equation (12)	Overall prediction correctness.	General reference metric.
Precision	Equation (13)	Reliability of predicted failures.	Reduces unnecessary maintenance actions caused by false alarms.
Recall	Equation (14)	Ability to detect actual failures.	Safety-critical indicator for PdM.
F1-score	Equation (15)	Balances Precision and Recall.	Overall assessment under class imbalance.
Balanced Accuracy	Equation (16)	Balances prediction performance across both classes.	Fair comparison under severe class imbalance.
MCC	–	Provides a balanced evaluation by jointly considering TP, TN, FP, and FN under class imbalance.	Recommended deployment metric for imbalanced PdM applications.

The deployment-oriented evaluation described above completes the proposed Physics-Informed Benchmarking Framework. By integrating physics-informed feature engineering, feature relevance validation, standardized benchmark model development, and comprehensive performance assessment, the proposed framework provides a systematic methodology for fair comparison among representative ML paradigms.

Collectively, the proposed methodology establishes a standardized and reproducible benchmark workflow in which representative ML paradigms can be objectively evaluated using identical feature representations, preprocessing procedures, and deployment-oriented assessment criteria. The experimental results obtained using this unified methodology are presented in the following section.

## 4. Results and Discussion

This section presents the experimental results obtained using the proposed Physics-Informed Benchmarking Framework. Rather than directly comparing the benchmark ML models, the analysis first examines the statistical characteristics of the proposed feature representation, followed by feature relevance validation, benchmark performance comparison, deployment-oriented evaluation, and engineering interpretation. This progressive evaluation strategy directly corresponds to the methodology presented in Section 3 and systematically validates each component of the proposed framework.

### 4.1. Statistical Characteristics of the Physics-Informed Feature Representation

The first objective of the experimental analysis is to examine whether the proposed physics-informed feature engineering strategy effectively enriches the original AI4I sensor measurements. Instead of evaluating predictive performance directly, this section investigates how engineering-guided feature construction enhances the original feature representation by incorporating physically meaningful descriptors associated with thermal behaviour, mechanical loading, feature interactions, and degradation evolution. These statistical observations provide the foundation for the subsequent Mutual Information (MI)-based feature relevance analysis presented in Section 4.2.

**Figure 11:**
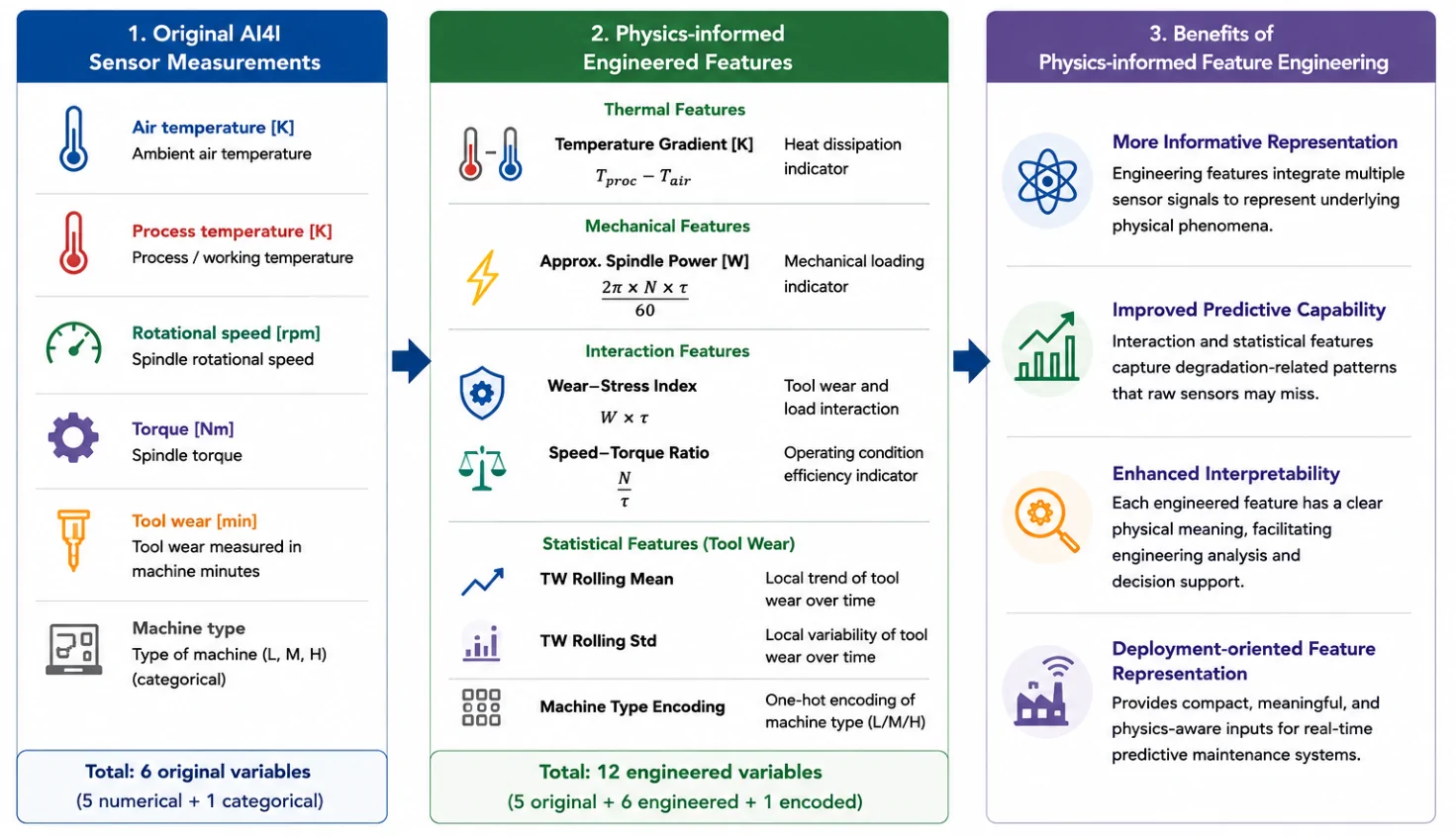
Comparison of the original AI4I sensor measurements and the proposed physics-informed engineered feature set. Engineering-derived features integrate thermal, mechanical, interaction, and statistical information to enrich the validated feature set while preserving physical interpretability. The resulting feature space provides the basis for the subsequent Mutual Information-based feature relevance analysis.

Figure 11 compares the original AI4I sensor measurements with the proposed physics-informed feature space. The original dataset contains five numerical sensor variables together with one categorical machine descriptor, whereas the proposed methodology enriches the feature space by introducing engineering-derived thermal, mechanical, interaction, and statistical features constructed according to established physical degradation mechanisms.

Several important observations can be drawn from Figure 11. First, the proposed feature engineering strategy substantially enriches the original engineered feature set by introducing physically meaningful variables that explicitly characterize thermal conditions, mechanical loading, interaction effects, and local degradation behaviour. Unlike the original sensor measurements, which independently describe individual operating conditions, the engineered features explicitly capture coupled physical relationships that more closely reflect practical equipment degradation mechanisms.

Second, the engineered interaction features provide complementary information that cannot be directly inferred from individual sensor variables. For example, the temperature gradient represents heat dissipation characteristics, approximate spindle power reflects spindle loading conditions, while the wear–stress index and speed–torque ratio describe interactions between operational variables associated with degradation progression. In addition, rolling statistical features characterize local wear evolution by incorporating temporal variations in tool wear.

Table 8 summarizes the composition of the original and the proposed physics-informed feature spaces adopted throughout the benchmark experiments.

As summarized in Table 8, the proposed methodology expands the original six input variables into twelve physically interpretable features while preserving the complete set of original sensor measurements. Rather than increasing feature dimensionality through arbitrary mathematical transformations, each engineered variable is explicitly designed according to engineering principles associated with equipment degradation. Consequently, the resulting feature space simultaneously enhances physical interpretability and provides a richer analytical basis for PdM modeling.

Although the statistical comparison confirms that the proposed feature engineering strategy enriches the original engineered feature set, descriptive observations alone cannot quantify the predictive contribution of individual features. Therefore, the following section employs Mutual Information (MI) analysis to objectively evaluate the relevance of both the original sensor variables and the engineered features before benchmark model development.

### 4.2. Mutual Information Analysis

Although the statistical comparison presented in Section 4 demonstrates that the proposed physics-informed feature engineering strategy enriches the feature space, descriptive observations alone cannot determine the predictive contribution of individual features. Therefore, MI analysis is employed to quantitatively evaluate the relevance of both the original sensor variables and the engineered features with respect to machine failure. By measuring nonlinear statistical dependency without assuming linear relationships, MI provides an objective criterion for assessing the information contribution of each feature before benchmark model development.

**Figure 12:**
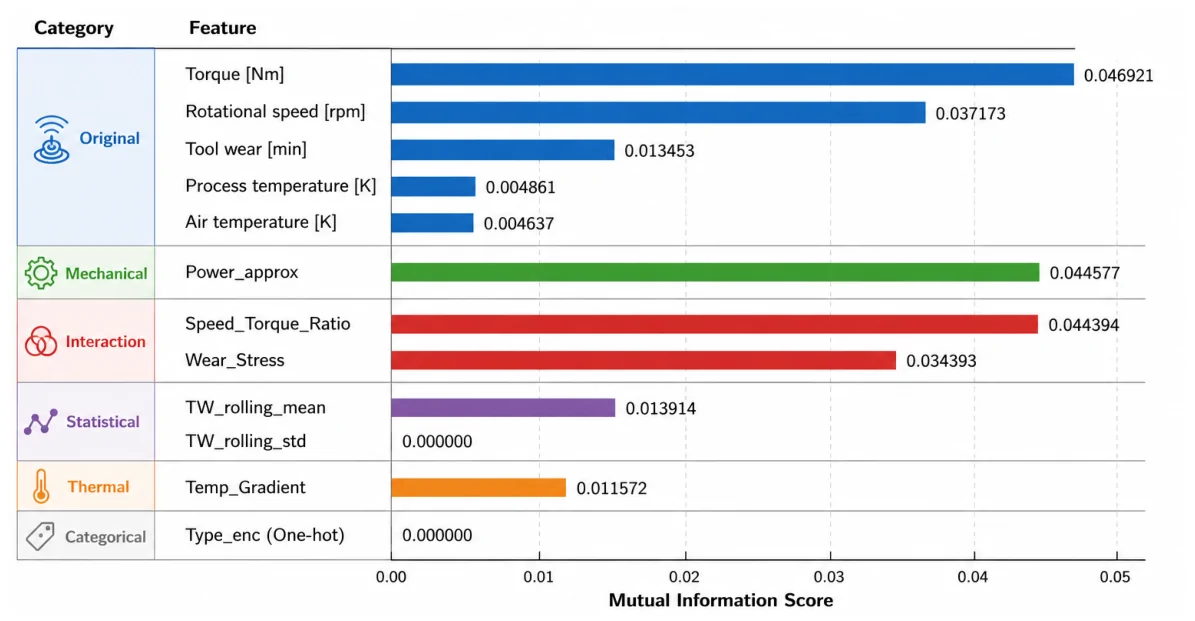
MI scores of the original sensor variables and the proposed physics-informed engineered features. The engineering-derived variables, particularly the mechanical and interaction features, exhibit high predictive relevance while complementing the information contained in the original AI4I sensor measurements.

Figure 12 presents the normalized MI scores of the original sensor variables and the proposed physics-informed engineered features. For clarity, the features are grouped according to their engineering categories, allowing direct comparison of their relative predictive relevance.

Several important observations can be drawn from Figure 12. First, both the original sensor variables and the proposed physics-informed engineered features are distributed among the highest-ranked features, indicating that the proposed methodology enriches rather than replaces the original AI4I input representation.

Second, the mechanical feature (Approximate Spindle Power) and the interaction features (Speed–Torque Ratio and Wear–Stress Index) achieve MI scores comparable to those of the original Torque measurement. These observations indicate that engineering-guided feature construction successfully captures degradation-related information beyond individual sensor observations.

Furthermore, the thermal and statistical features provide complementary predictive information associated with heat dissipation and local degradation evolution. Although their MI scores are comparatively lower, these features remain physically meaningful and contribute additional engineering insight for PdM.

To facilitate quantitative comparison, Table 9 summarizes the MI scores together with the engineering interpretation of the original sensor variables and the proposed physics-informed engineered features. The ranking provides an objective assessment of the predictive relevance of each feature while preserving the associated engineering interpretation of each feature.

As shown in Table 9, the top-ranked features comprise both raw sensor readings and engineered variables, indicating that the proposed feature engineering approach supplements rather than replicates the information in the original AI4I dataset. Notably, the Approximate Spindle Power and the Speed–Torque Ratio achieve MI scores similar to the original Torque feature, suggesting that physically interpretable combinations of multiple sensor signals offer additional predictive value for machine failure forecasting.

Moreover, the Wear–Stress Index and TW Rolling Mean further characterize degradation interactions and wear progression that cannot be directly represented by individual sensor variables. Although the Temperature Gradient exhibits a comparatively lower MI score, it remains valuable because it explicitly represents heat dissipation characteristics and improves the physical interpretability of the feature space. Overall, the MI analysis quantitatively validates the engineering rationale underlying the proposed feature engineering strategy and confirms that the resulting validated feature set provides an informative and physically interpretable input space for benchmark model development.

Notably, the MI analysis is intended to validate the engineering rationale of the constructed feature space rather than to perform aggressive feature selection. Consequently, features with moderate MI scores may still be retained when they represent important physical degradation mechanisms or provide valuable engineering insight for maintenance decision-making.

Although MI evaluates the predictive relevance of individual features independently, the practical effectiveness of the proposed input representation ultimately depends on how different benchmark ML models exploit these informative variables. Therefore, the following section compares representative learning paradigms developed under the unified Physics-Informed Benchmarking Framework to evaluate the practical impact of the validated feature space on PdM performance.

### 4.3. Benchmark Performance Comparison

Following the validation of feature relevance using MI analysis, benchmark experiments were conducted to evaluate how effectively representative ML algorithms exploit the validated feature representation. Rather than identifying a universally optimal classifier, this benchmark aims to establish a fair and reproducible comparison among representative learning paradigms under an identical experimental protocol.

All benchmark models were trained and evaluated using the same training–testing partition, preprocessing procedures, feature representation, class imbalance treatment, and hyperparameter configuration described in Section 3.5. Consequently, the performance differences reported in this section primarily reflect the intrinsic learning characteristics of the algorithms rather than inconsistencies in experimental settings.

**Figure 13:**
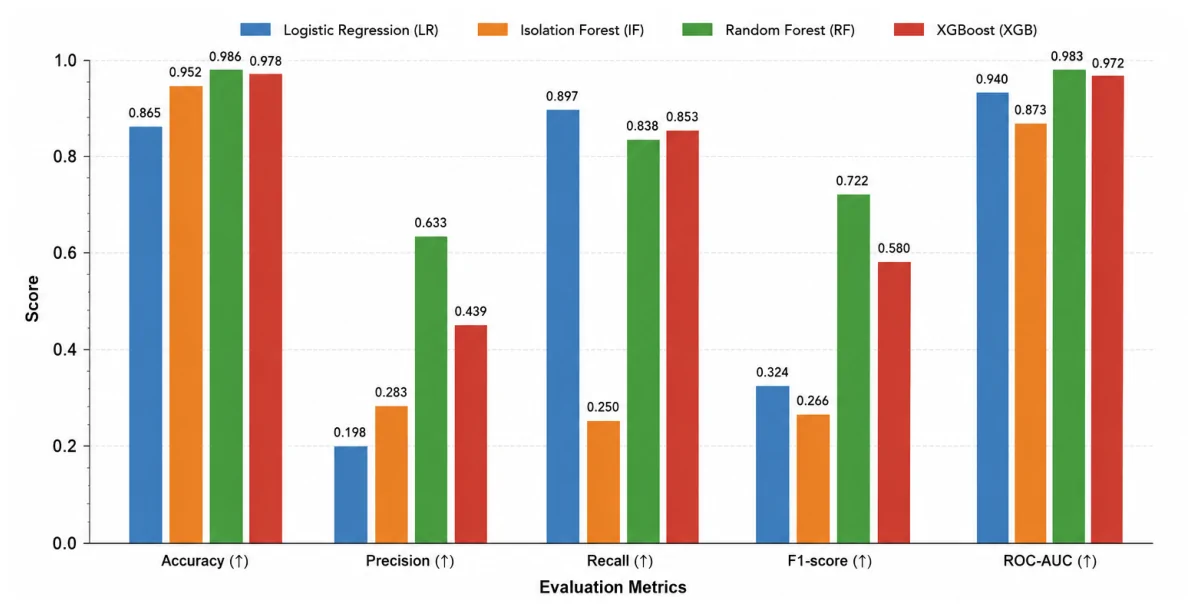
Performance comparison of representative benchmark machine learning algorithms using the proposed physics-informed feature representation. Multiple deployment-oriented evaluation metrics are adopted to provide a comprehensive assessment under severe class imbalance.

Figure 13 compares the predictive performance of the representative benchmark ML models using the proposed physics-informed feature representation. Multiple deployment-oriented evaluation metrics, including Accuracy, Precision, Recall, F1-score, and ROC-AUC, are employed to provide a comprehensive assessment of benchmark performance, while MCC is additionally adopted to evaluate classifier robustness under severe class imbalance.

Several important observations can be obtained from Figure 13. First, the tree-based ensemble models consistently outperform the linear and unsupervised approaches across most evaluation metrics. In particular, XGBoost and Random Forest achieve the highest overall predictive performance, indicating their capability to effectively utilize the validated physics-informed feature representation.

Second, the performance differences become substantially more pronounced when deployment-oriented evaluation metrics are considered. Although several models exhibit comparable overall accuracy, larger discrepancies are observed in Recall, F1-score, and ROC-AUC, indicating that conventional accuracy alone is insufficient for evaluating PdM models under highly imbalanced industrial conditions.

To facilitate quantitative comparison, Table 10 summarizes the benchmark performance of the representative ML models using the proposed physics-informed feature representation.

As summarized in Table 10, the tree-based ensemble models consistently outperform the linear and unsupervised approaches across multiple evaluation metrics. Random Forest achieves the highest overall classification performance, whereas XGBoost provides the lowest inference latency while maintaining competitive predictive capability. These results demonstrate that both ensemble models effectively exploit the validated physics-informed feature representation.

Beyond predictive performance, Random Forest provides additional engineering interpretability through feature importance analysis. Logistic Regression serves as an interpretable linear baseline, whereas Isolation Forest remains applicable when labeled failure data are unavailable.

The benchmark comparison further demonstrates that the observed performance improvements cannot be solely attributed to increasing feature dimensionality. Instead, the engineering-guided feature construction validated through Mutual Information analysis provides physically meaningful representations that can be effectively exploited by different learning paradigms. Consequently, the benchmark results confirm that engineering-guided feature construction improves the effectiveness of representative ML models while establishing a standardized and reproducible benchmark for deployment-oriented PdM.

To further investigate whether the observed performance improvement originates from the proposed physics-informed feature engineering rather than from classifier-specific characteristics, a feature ablation study was conducted.

**Figure 14:**
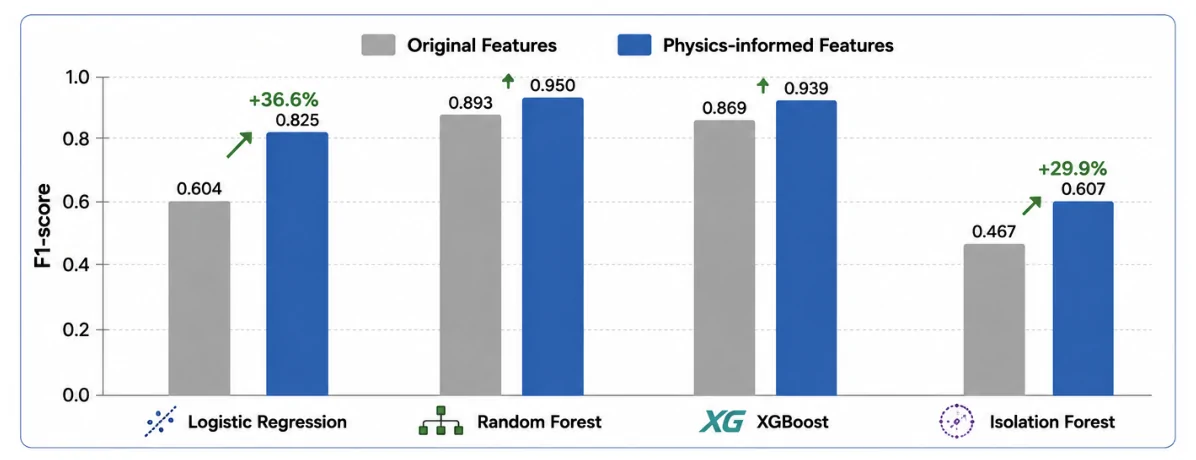
Feature ablation analysis comparing benchmark models trained using the original sensor variables and the proposed physics-informed feature set. The consistent F1-score improvement across different learning paradigms demonstrates the effectiveness of the proposed engineering-guided feature construction.

Figure 14 demonstrates that all representative benchmark models benefit from the proposed physics-informed features. Although the magnitude of improvement varies among classifiers, consistent F1-score gains are observed for Logistic Regression, Random Forest, XGBoost, and Isolation Forest. These results indicate that the observed performance improvement primarily results from the proposed physics-informed feature representation rather than from classifier-specific learning characteristics.

Given the severe class imbalance of the AI4I dataset, MCC was additionally adopted to provide a more balanced evaluation of classifier robustness. Unlike Accuracy or F1-score, MCC simultaneously considers true positives, true negatives, false positives, and false negatives, providing a more balanced evaluation under imbalanced industrial datasets.

**Figure 15:**
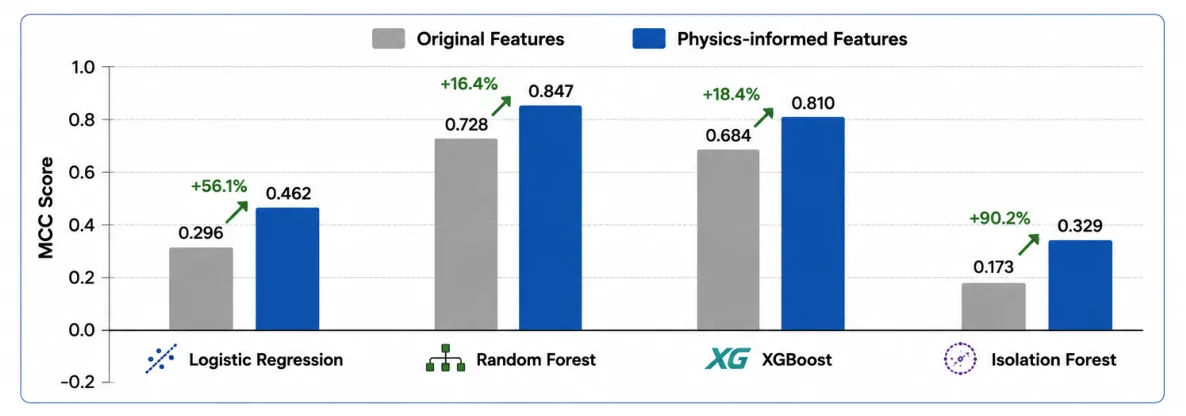
Comparison of MCC obtained using the original feature set and the proposed physics-informed feature set. Consistent MCC improvement demonstrates enhanced robustness under severe class imbalance.

As illustrated in Figure 15, all benchmark models exhibit higher MCC values after incorporating the proposed physics-informed features. The consistent improvement indicates that the proposed feature engineering strategy not only improves predictive performance but also enhances classification robustness under realistic industrial class imbalance, thereby providing complementary evidence for the robustness and generalizability of the proposed Physics-Informed Benchmarking Framework.

Although the overall benchmark comparison demonstrates the effectiveness of the proposed framework, practical industrial deployment requires more detailed evaluation of failure detection capability and prediction robustness than can be inferred from aggregate performance metrics alone. Therefore, the following section further analyzes confusion matrices together with deployment-oriented performance metrics to assess the practical applicability of the representative benchmark models.

### 4.4. Deployment-Oriented Benchmark Results

Although the benchmark comparison in Section 4.3 highlights the overall predictive performance of the selected ML models, practical industrial deployment demands a more extensive evaluation than overall classification accuracy alone. In PdM settings, accurately detecting imminent equipment failures is far more critical than maximizing the rate of correctly classified normal operating states. Consequently, this section further analyzes the benchmark models using deployment-oriented evaluation criteria that explicitly incorporate the effects of severe class imbalance and the requirements of maintenance decision-making.

**Figure 16:**
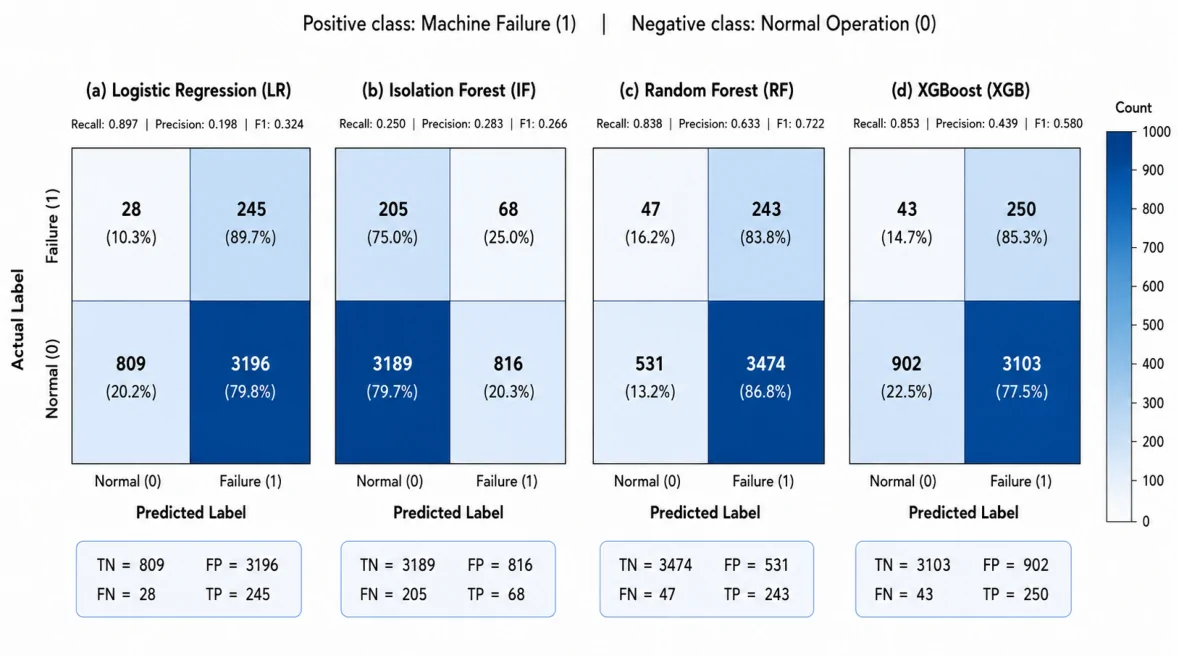
Comparison of the confusion matrices obtained by the representative benchmark machine learning algorithms. The confusion matrices illustrate the distribution of true-positive, true-negative, false-positive, and false-negative predictions under identical experimental conditions, thereby providing deployment-oriented insight into model reliability.

Figure 16 presents the confusion matrices for representative benchmark ML models constructed using the proposed Physics-Informed Benchmarking Framework. In contrast to aggregate performance metrics, confusion matrices directly display how correct and incorrect predictions are distributed, thereby offering more detailed insight into the models’ behaviour in practical deployment.

Several important observations can be obtained from Figure 16. First, the tree-based ensemble algorithms substantially reduce false-negative predictions compared with the linear and unsupervised approaches. Because false-negative predictions correspond to undetected equipment failures, minimizing this error type is particularly important for PdM applications.

Second, although all benchmark models correctly classify most normal operating conditions owing to the severe class imbalance of the AI4I dataset, their capability to identify failure samples differs considerably. This finding further confirms that overall accuracy alone cannot adequately reflect deployment performance under realistic industrial operating conditions.

Table 11 summarizes the deployment-oriented assessment of the representative benchmark ML models from the perspectives of failure detection capability, false-alarm control, maintenance cost, and deployment suitability. Unlike the quantitative performance comparison presented in Table 10, this qualitative assessment emphasizes engineering decision-making under realistic industrial operating conditions.

As summarized in Table 11, deployment suitability is determined not only by predictive accuracy but also by the trade-offs among failure detection capability, false-alarm management, maintenance burden, computational efficiency, and practical engineering usability. While Logistic Regression achieves very high sensitivity to failures, its comparatively weak control of false alarms can lead to avoidable maintenance actions. Isolation Forest is a practical choice for anomaly detection when labeled failure data are not available; however, its relatively low failure detection capability limits its appropriateness for deployment-focused PdM.

Among the benchmarked models, Random Forest offers the most well-rounded balance between predictive performance and engineering interpretability, whereas XGBoost demonstrates the highest overall deployment potential due to its superior predictive accuracy, low inference latency, and computational efficiency. Therefore, XGBoost is recommended for real-time IIoT-based PdM, with Random Forest remaining a compelling option for use cases in which model transparency and engineering interpretability are of primary importance.

These deployment-oriented observations demonstrate that model selection should be guided not only by predictive accuracy but also by the practical operational requirements of industrial maintenance systems.

### 4.5. Model Interpretability and Engineering Insights

While the benchmark experiments indicate strong predictive performance, practical application in industrial settings also demands models that are transparent and interpretable. In addition to accurate failure prediction, maintenance engineers must be able to identify which physical degradation mechanisms influence the model’s decisions, both to guide maintenance planning and to increase trust in predictive analytics. Consequently, this section examines the engineering interpretation of the benchmark findings through the lens of the proposed physics-informed feature representation.

The benchmark evaluation presented in Section 4.3 and Section 4.4 indicates that tree-based ensemble learning algorithms consistently outperform the linear and unsupervised approaches across multiple deployment-oriented performance metrics. This superior predictive capability is closely associated with the ability of ensemble models to exploit nonlinear interactions among heterogeneous IIoT sensor variables. Since the proposed feature engineering strategy explicitly incorporates engineering knowledge into the feature representation, the resulting benchmark models retain physical interpretability while maintaining high predictive performance.

The experimental results indicate that the proposed physics-informed engineered features effectively complement the original AI4I sensor measurements by explicitly representing different degradation mechanisms. Mechanical features, such as the Approximate Spindle Power, characterize spindle loading conditions by integrating rotational speed and torque. Interaction features, including the Wear–Stress Index and Speed–Torque Ratio, capture coupled degradation behaviour that cannot be represented by individual sensor variables alone. In addition, statistical features describe the local evolution of tool degradation, while thermal features characterize heat dissipation during machining operations. Together, these engineering-derived variables provide a more comprehensive representation of machine health than the original sensor measurements alone.

**Figure 17:**
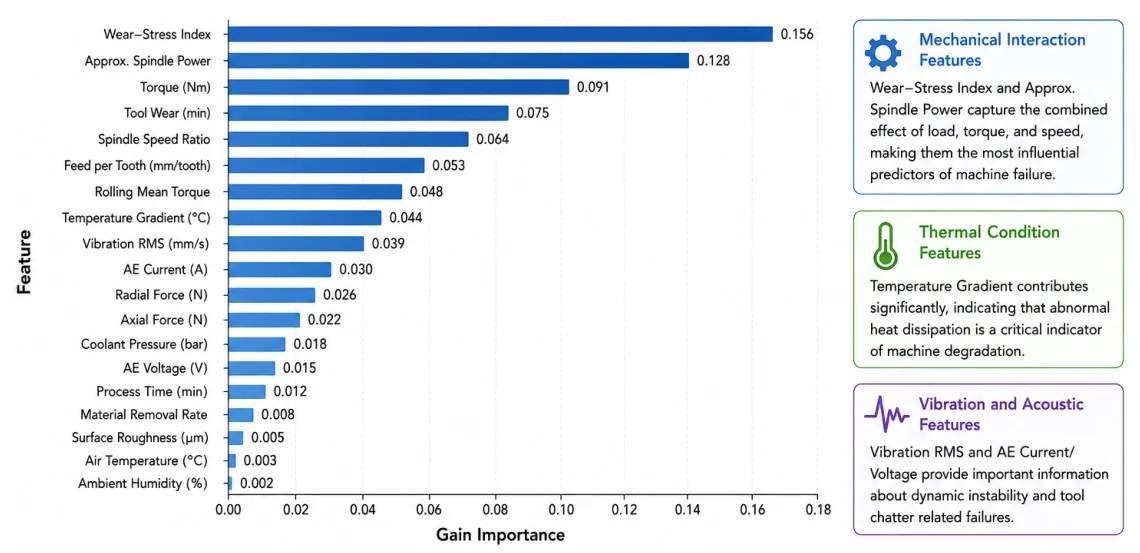
Feature importance obtained from the XGBoost model using the proposed physics-informed feature representation. Engineering-derived interaction features, particularly the Wear–Stress Index and Approximate Spindle Power, exhibit the highest predictive contribution, demonstrating the effectiveness of incorporating engineering knowledge into feature construction.

Figure 17 presents the feature importance derived from the XGBoost model using the validated feature representation. In particular, the Wear–Stress Index and Approximate Spindle Power exhibit the highest importance scores, supporting that interaction-based variables effectively capture coupled degradation mechanisms that cannot be adequately represented by individual sensor measurements.

These observations further explain why the tree-based ensemble models consistently outperform the linear baseline, as they are better able to capture nonlinear interactions embedded in the proposed physics-informed feature representation.

The engineering interpretation is further supported by the MI analysis presented in Section 4.2. Features exhibiting relatively high MI scores are primarily associated with mechanical loading and degradation interactions, indicating that these physical mechanisms provide the greatest predictive information for machine failure. The consistency between statistical feature relevance and benchmark model performance demonstrates that the proposed feature engineering strategy successfully captures physically meaningful degradation characteristics rather than introducing redundant variables.

Unlike purely data-driven feature extraction approaches, the proposed methodology preserves explicit engineering interpretation throughout the entire benchmarking process. Each engineered feature corresponds to a well-defined physical mechanism associated with equipment degradation, enabling maintenance engineers to understand the rationale behind model predictions. This interpretability is particularly important for industrial PdM, where maintenance decisions often require both high predictive accuracy and model transparency.

To further investigate the physical significance of the benchmark models, the engineering interpretation of the most influential features is summarized in Table 12. Rather than reporting feature importance values alone, the present analysis relates each important variable to its underlying physical mechanism and the corresponding failure mode represented in the AI4I dataset.

As summarized in Table 12, the most influential features not only correspond to the principal degradation mechanisms represented in the AI4I dataset but also provide actionable maintenance insights for industrial decision-making. The engineering-derived interaction, mechanical, thermal, and statistical features collectively enable maintenance engineers to better understand machine health conditions and prioritize appropriate maintenance actions.

From an industrial perspective, the benchmark results indicate that machine degradation in the AI4I dataset is primarily governed by mechanical loading conditions together with their interaction with accumulated tool wear. Thermal descriptors provide complementary information by characterizing heat dissipation behaviour, whereas statistical features describe local degradation evolution over time. These observations are consistent with established engineering knowledge regarding machining systems and therefore strengthen confidence in the practical applicability of the proposed Physics-Informed Benchmarking Framework.

In summary, the interpretability analysis demonstrates that the proposed framework not only improves predictive performance but also preserves meaningful engineering interpretation throughout the learning process. By integrating engineering knowledge into feature construction and validating feature relevance before benchmark model development, the proposed methodology establishes an interpretable and deployment-oriented PdM framework suitable for IIoT-enabled smart manufacturing. The broader implications of these findings, together with the limitations of the proposed methodology, are discussed in the following section.

### 4.6. Discussion

The experimental findings collectively demonstrate the effectiveness of the proposed Physics-Informed Benchmarking Framework for deployment-oriented PdM. Unlike conventional benchmarking studies that primarily compare the predictive performance of different ML models, the proposed framework integrates physics-informed feature engineering, feature relevance validation, standardized benchmark model development, and deployment-oriented evaluation into a unified experimental methodology. Consequently, it establishes a standardized and reproducible benchmarking framework that enables objective algorithm comparison while preserving engineering interpretability.

A key contribution of this study is the systematic integration of engineering knowledge into the benchmark design. Rather than depending solely on raw sensor signals, the proposed approach generates physics-informed engineered features that capture thermal dynamics, spindle load conditions, degradation interactions, and statistical wear progression. Mutual Information analysis verifies that these engineered features provide substantial predictive information, and the benchmark experiments show that widely used machine learning models can effectively leverage this enriched feature space. This integrated strategy clearly differentiates the proposed framework from purely data-driven benchmarking approaches.

The additional feature ablation and MCC analyses further reinforce this observation. The consistent performance improvements achieved across representative machine learning models indicate that the proposed physics-informed feature representation contributes to enhanced predictive capability independently of classifier selection. Moreover, the improved MCC values under severe class imbalance demonstrate that the proposed feature engineering strategy enhances not only predictive performance but also the robustness and reliability of deployment-oriented PdM. This finding is also consistent with previous studies demonstrating that incorporating domain knowledge into machine learning frameworks can improve predictive performance by guiding feature representation and model design rather than relying solely on purely data-driven learning [47].

The benchmark comparison further indicates that tree-based ensemble models consistently surpass linear and unsupervised methods within the unified experimental setting. In particular, Random Forest and XGBoost deliver the best predictive performance by capturing nonlinear dependencies among heterogeneous IIoT sensor measurements while maintaining robustness under pronounced class imbalance. At the same time, Logistic Regression remains useful as a transparent, interpretable baseline, and Isolation Forest offers a viable alternative for use cases in which labeled failure data are not available.

From an industrial standpoint, the proposed framework offers practical benefits that extend beyond predictive accuracy alone. The deployment-oriented evaluation indicates that maintenance decisions should prioritize failure detection capabilities in combination with balanced predictive performance, rather than relying solely on overall classification accuracy. In addition, the engineering-based interpretation of the constructed features allows maintenance engineers to understand the underlying physical mechanisms driving the model outputs, thereby enhancing transparency and trust in PdM solutions.

Nevertheless, several limitations need to be recognized. First, the experimental analysis relies on the publicly available AI4I 2020 dataset, which is a synthetic benchmark designed to emulate realistic industrial operating conditions. While this benchmark supports reproducible comparisons with prior work, its synthetic nature cannot fully capture the variability, noise characteristics, equipment heterogeneity, and operational uncertainties encountered in real manufacturing environments. Further validation on real-world industrial datasets from diverse manufacturing processes would strengthen the external validity and robustness of the proposed framework.

Future investigations can broaden the scope of the proposed Physics-Informed Benchmarking Framework in several ways. A key opportunity lies in integrating domain adaptation strategies to enhance model generalization across diverse and multi-sensor manufacturing settings. Another promising direction is the inclusion of explainable artificial intelligence techniques, such as SHAP or LIME, to increase model interpretability and better support engineering decision-making. Moreover, adapting the framework to handle streaming IIoT sensor data and online PdM constitutes a crucial step toward achieving real-time industrial implementation.

In summary, the proposed Physics-Informed Benchmarking Framework provides a standardized methodology for integrating engineering knowledge into feature construction, feature relevance validation, benchmark model development, and deployment-oriented evaluation. By combining physics-informed feature engineering with representative ML paradigms under a unified experimental protocol, the framework enhances predictive performance while preserving engineering interpretability and practical deployment applicability. Consequently, this work establishes a standardized and reproducible benchmarking framework that provides a solid foundation for future research and real-world implementation of IIoT-enabled PdM systems.

Beyond PdM, the proposed benchmarking methodology may also be extended to other industrial condition monitoring and intelligent manufacturing applications that require physically interpretable feature representations and standardized model evaluation.

## 5. Conclusions

This study proposed a Physics-Informed Benchmarking Framework for deployment-oriented PdM. The framework integrates physics-informed feature engineering, feature relevance validation, benchmark model development, and deployment-oriented evaluation into a unified and reproducible experimental methodology. Using the publicly available AI4I 2020 Predictive Maintenance Dataset, the proposed framework establishes a standardized benchmark for objectively comparing representative ML paradigms under identical experimental conditions.

The experimental results demonstrate that the proposed physics-informed feature representation effectively enriches the original AI4I sensor measurements by incorporating thermal, mechanical, interaction, and statistical degradation characteristics. Mutual Information analysis, benchmark experiments, feature ablation analysis, and MCC evaluation consistently confirm that the engineering-derived features provide meaningful predictive information while preserving physical interpretability. Among the evaluated benchmark models, Random Forest achieves the best overall predictive performance, whereas XGBoost provides the most favorable balance between predictive capability and computational efficiency for deployment-oriented IIoT applications.

The additional feature ablation and MCC analyses further demonstrate that the proposed physics-informed feature representation consistently improves predictive performance and classification robustness across representative machine learning models, reinforcing the general applicability of the proposed benchmarking framework under highly imbalanced industrial conditions.

The principal contribution of this work lies in establishing a standardized benchmarking methodology rather than proposing a new prediction algorithm. By integrating engineering knowledge into feature construction and evaluating representative ML models under a unified experimental protocol, the proposed framework enables fair comparison across different learning paradigms while supporting engineering interpretation and practical deployment.

Several limitations should be acknowledged. Although the AI4I 2020 Predictive Maintenance Dataset provides a standardized and reproducible benchmark for objective evaluation, it is a synthetic dataset designed to emulate representative industrial operating conditions. Consequently, the experimental results may not fully capture the complexity, uncertainty, and operational variability encountered in real manufacturing environments. In addition, the present study focuses on representative lightweight ML models under a unified benchmarking protocol. Future research will extend the proposed framework to real-world industrial datasets, streaming IIoT sensor data, explainable AI techniques, digital twin environments, and foundation models for PdM to further validate its applicability and generalizability across diverse industrial scenarios.

Overall, the proposed Physics-Informed Benchmarking Framework provides a standardized, reproducible, and interpretable methodology for deployment-oriented PdM. By integrating engineering knowledge with representative ML paradigms under a unified experimental protocol, this work establishes a practical foundation for future research and real-world implementation of intelligent maintenance systems in IIoT-enabled smart manufacturing.

## Figures and Tables

**Table 1:** Comparison of representative machine learning paradigms for PdM.

Learning Paradigm	Representative Algorithm	Major Strengths	Primary Limitations	Deployment Characteristics
Linear Model	Logistic Regression	Simple implementation, high interpretability, fast training and inference.	Limited capability for modeling nonlinear relationships.	Baseline failure classification.
Tree-based Ensemble	Random Forest	Robust to noise, models nonlinear interactions, provides feature importance.	Larger model size and reduced interpretability compared with linear models.	General industrial PdM.
Tree-based Ensemble	XGBoost	Excellent predictive accuracy, strong generalization capability, and handles heterogeneous features.	Requires hyperparameter tuning and higher computational cost during training.	High-performance PdM benchmarking.
Unsupervised Learning	Isolation Forest	Does not require failure labels and performs well for anomaly detection.	May exhibit lower discrimination when sufficient labeled failure data are available.	Early anomaly detection.
Deep Learning	CNN, LSTM, Transformer	Automatically learns hierarchical feature representations from large-scale sensor data.	Requires extensive training data, high computational resources, and offers limited interpretability.	Large-scale sequential IIoT monitoring.

**Table 2:** Comparison of representative predictive maintenance studies based on key methodological components.

Reference	Physics-Informed Feature	Feature Relevance	Benchmark Protocol	Deployment Evaluation
Carvalho et al. [19]	○	○	○	○
Zonta et al. [9]	○	○	○	○
Serradilla et al. [21]	○	○	○	○
Nsor et al. [32]	•	•	○	○
This Work	•	•	•	•

• Explicitly addressed as a primary methodological component. ○ Not explicitly addressed as a primary methodological component.

**Table 3:** Summary of Variables in the AI4I 2020 Predictive Maintenance Dataset.

Variable	Unit	Type	Description	Role in the Proposed Framework
Machine Type	–	Categorical	Machine quality category (L, M, H).	Characterizes machine operating conditions and production quality.
Air Temperature	K	Continuous	Ambient temperature surrounding the machine.	Reference variable for thermal feature construction.
Process Temperature	K	Continuous	Temperature measured during machining.	Combined with air temperature to derive thermal-related features.
Rotational Speed	rpm	Continuous	Spindle rotational speed.	Used to construct power-related and mechanical loading features.
Torque	Nm	Continuous	Applied spindle torque.	Combined with rotational speed to estimate spindle power and mechanical load.
Tool Wear	min	Continuous	Accumulated tool operating time.	Primary degradation variable for wear-related feature construction.
Failure Label	Binary	Target	Normal (0) or Failure (1).	Target variable for benchmark model training and evaluation.
Failure Mechanism	–	Categorical	TWF, HDF, PWF, OSF, and RNF.	Used for failure mechanism interpretation but excluded from binary classification.

**Table 4:** Definitions and Engineering Interpretation of the Proposed Physics-informed Features.

Category	Engineered Feature	Equation	Original Variables	Physical Interpretation	Expected Contribution
Thermal	Temperature Gradient	Equation (1)	Process Temperature, Air Temperature	Represents the thermal difference between the machining process and the surrounding environment.	Improves sensitivity to heat accumulation and thermal-related failures (e.g., HDF).
Mechanical	Approximate Spindle Power	Equation (2)	Torque, Rotational Speed	Represents mechanical loading by combining spindle torque and rotational speed.	Provides a physically meaningful indicator of machine operating load.
Interaction	Wear–Stress Index	Equation (3)	Tool Wear, Torque	Measures the combined effect of accumulated wear and mechanical loading.	Enhances discrimination of progressive degradation under heavy loading conditions.
Interaction	Speed–Torque Ratio	Equation (4)	Rotational Speed, Torque	Characterizes operational efficiency by comparing rotational output with applied load.	Provides complementary information beyond individual sensor measurements.
Statistical	Rolling Mean of Tool Wear	Equation (5)	Tool Wear	Represents the local average wear condition over neighboring production samples.	Captures degradation trend while reducing local fluctuations.
Statistical	Rolling Standard Deviation of Tool Wear	Equation (6)	Tool Wear	Quantifies the local variability of tool wear.	Improves sensitivity to unstable degradation behavior prior to failure.
Categorical	Encoded Machine Type	Equation (7)	Machine Type	Transforms categorical production information into numerical representation.	Allows machine quality information to participate in machine learning modeling.

**Table 5:** Mutual Information-Based Feature Relevance Analysis Protocol.

Evaluation Item	Description
Analysis Method	Mutual Information (MI)
Feature Set	Original sensor variables and engineered features
Target Variable	Binary failure label
Dependency Type	Linear and nonlinear statistical relationships
Selection Principle	Higher MI values indicate greater predictive relevance
Evaluation Purpose	Quantify feature relevance before benchmark model development
Output	Ranked feature relevance scores for benchmark feature validation

**Table 6:** Summary of widely used benchmark ML models and their fixed experimental configurations within the proposed Physics-Informed Benchmarking Framework. The reported hyperparameter values correspond to the fixed benchmark configurations adopted throughout all comparative experiments.

Model	Representative Hyperparameters	Configuration	Purpose in Benchmark
Logistic Regression (LR)	Penalty, Solver, Max Iterations	L2 regularization, lbfgs solver, max_iter = 1000	Interpretable linear baseline model used to evaluate the effectiveness of physics-informed features.
Isolation Forest (IF)	Number of Trees, Contamination, Random State	n_estimators = 100, contamination = auto, random_state = 42	Representative unsupervised anomaly detection algorithm for highly imbalanced PdM.
Random Forest (RF)	Number of Trees, Maximum Depth, Random State	n_estimators = 200, max_depth = None, random_state = 42	Bagging-based ensemble learner providing robust prediction and feature importance analysis.
Extreme Gradient Boosting (XGBoost)	Number of Trees, Learning Rate, Maximum Depth, Subsample	n_estimators = 200, learning_rate = 0.10, max_depth = 6, subsample = 0.8	Gradient boosting ensemble representing state-of-the-art supervised learning for tabular industrial data.

**Table 8:** Composition of the original and physics-informed feature representations used for benchmark model development.

Feature Category	Original	Physics-Informed Representation
Numerical Sensor Variables	5	5
Categorical Variables	1	1 (One-hot encoded)
Thermal Features	–	Temperature Gradient
Mechanical Features	–	Approximate Spindle Power
Interaction Features	–	Wear–Stress Index
		Speed–Torque Ratio
Statistical Features	–	TW Rolling Mean
		TW Rolling Std
Total Features	**6**	**12**

**Table 9:** MI ranking and engineering interpretation of the original sensor variables and the proposed physics-informed engineered features.

Rank	Feature	Category	MI Score	Engineering Interpretation
1	Torque	Original	0.0469	Direct indicator of spindle loading and cutting resistance.
2	Approximate Spindle Power	Mechanical	0.0446	Combines rotational speed and torque to estimate spindle mechanical loading.
3	Speed–Torque Ratio	Interaction	0.0444	Represents the operating efficiency and balance between spindle speed and applied load.
4	Rotational Speed	Original	0.0372	Describes spindle operating conditions during machining.
5	Wear–Stress Index	Interaction	0.0344	Captures the coupled degradation effect between accumulated tool wear and spindle loading.
6	TW Rolling Mean	Statistical	0.0139	Represents the local degradation trend of tool wear.
7	Tool Wear	Original	0.0135	Primary cumulative degradation indicator of cutting tools.
8	Temperature Gradient	Thermal	0.0116	Characterizes heat dissipation between process and ambient temperatures.
9	Process Temperature	Original	0.0049	Reflects machining thermal conditions.
10	Air Temperature	Original	0.0046	Reference environmental operating condition.
11	TW Rolling Std	Statistical	0.0000	Represents local variability of tool wear.
12	Machine Type (One-hot)	Categorical	0.0000	Encodes production quality level (L/M/H).

**Table 10:** Quantitative performance comparison of representative benchmark ML models developed under the proposed Physics-Informed Benchmarking Framework. The best performance for each evaluation metric is highlighted in bold.

Model	Accuracy	Precision	Recall	F1-Score	ROC-AUC	Latency (ms)
LR	0.865	0.198	**0.897**	0.324	0.940	0.61
IF	0.952	0.283	0.250	0.266	0.873	0.72
RF	**0.986**	**0.633**	0.838	**0.722**	**0.983**	2.51
XGBoost	0.978	0.439	0.853	0.580	0.972	**0.35**

**Table 11:** Deployment-oriented assessment of representative benchmark ML models. The qualitative ratings are derived from the quantitative benchmark results reported in Table 10, considering Recall, Precision, inference latency, and overall engineering applicability.

Model	Failure Detection	False-Alarm Control	Maintenance Cost	Deployment Suitability
LR	High	Low	High	Provides the highest failure sensitivity but generates excessive false alarms, making it more appropriate for safety-critical applications where missed failures are unacceptable.
IF	Low	Moderate	High	Suitable as an unsupervised anomaly detection baseline when labeled failure data are unavailable, although its overall predictive capability is insufficient for deployment-oriented PdM.
RF	High	High	Moderate	Achieves the best overall balance between failure detection, false-alarm control, predictive performance, and engineering interpretability, making it suitable for general industrial deployment.
XGBoost	High	Good	Low	Combines competitive predictive performance with the lowest inference latency, making it particularly suitable for real-time IIoT-enabled PdM applications.

Note: Failure detection is primarily assessed using Recall; false-alarm control is assessed using Precision; maintenance cost reflects the operational impact of missed failures and unnecessary maintenance actions; deployment suitability is determined by jointly considering predictive performance, inference latency, computational efficiency, robustness, and engineering interpretability. The qualitative ratings are derived from the quantitative benchmark results reported in Table 10.

**Table 12:** Engineering interpretation and maintenance insights for the most influential features identified by the XGBoost model.

Feature	Feature Category	Physical Interpretation	Maintenance Insight
Wear–Stress Index	Interaction	Represents the combined effect of accumulated tool wear and spindle loading.	Provides an early indication of progressive mechanical degradation and supports timely tool replacement before severe wear-induced failures occur.
Approximate Spindle Power	Mechanical	Estimates spindle loading by combining torque and rotational speed.	Helps identify overload operating conditions and supports preventive inspection of spindle and drive components.
Torque	Original Sensor	Measures instantaneous mechanical loading during machining.	Indicates abnormal cutting resistance and assists in detecting excessive machining loads.
Tool Wear	Original Sensor	Represents cumulative degradation of cutting tools.	Supports maintenance scheduling and replacement planning according to wear progression.
Speed–Torque Ratio	Interaction	Characterizes the relationship between spindle speed and mechanical loading.	Reveals inefficient operating conditions and potential mechanical imbalance during machining.
Temperature Gradient	Thermal	Represents the temperature difference between process and ambient conditions.	Helps identify abnormal heat dissipation caused by excessive friction, inadequate cooling, or thermal overload.
Rolling Mean of Tool Wear	Statistical	Describes the local degradation trend during continuous machining.	Supports continuous monitoring of degradation progression and maintenance planning.
Rolling Standard Deviation of Tool Wear	Statistical	Measures the local variability of wear progression.	Provides early warning of unstable degradation behaviour that may precede unexpected equipment failures.

Note: The engineering interpretations are derived from the feature importance analysis of the XGBoost model together with the validated physics-informed feature representation. The maintenance insights summarize how each influential feature can support practical maintenance decision-making in IIoT-enabled PdM systems.

## Data Availability

The experiments in this study were conducted using the publicly available AI4I 2020 Predictive Maintenance Dataset. To support the transparency and reproducibility of this research, Version 1.0 of the implementation of the proposed physics-informed feature engineering, benchmarking framework, and evaluation procedures is publicly available at the following GitHub repository: https://github.com/ottolan0728/physics-informed-pdm-benchmark (accessed on 2 July 2026). The repository provides the source code and documentation required to reproduce the experimental workflow presented in this study. The original dataset is not redistributed in the repository and should be obtained from its official source.

## Abbreviations

AI4I	AI4I 2020 Predictive Maintenance Dataset
IIoT	Industrial Internet of Things
PdM	Predictive Maintenance
ML	Machine Learning
LR	Logistic Regression
IF	Isolation Forest
RF	Random Forest
XGBoost	Extreme Gradient Boosting
MI	Mutual Information
MCC	Matthews Correlation Coefficient
ROC-AUC	Area Under the Receiver Operating Characteristic Curve
F1	F1-score
SHAP	SHapley Additive exPlanations
LIME	Local Interpretable Model-agnostic Explanations
